# The interaction of innate immune and adaptive immune system

**DOI:** 10.1002/mco2.714

**Published:** 2024-09-15

**Authors:** Ruyuan Wang, Caini Lan, Kamel Benlagha, Niels Olsen Saraiva Camara, Heather Miller, Masato Kubo, Steffen Heegaard, Pamela Lee, Lu Yang, Huamei Forsman, Xingrui Li, Zhimin Zhai, Chaohong Liu

**Affiliations:** ^1^ Department of Thyroid and Breast Surgery Tongji Hospital, Tongji Medical College, Huazhong University of Science and Technology Wuhan China; ^2^ Cancer Center Union Hospital, Tongji Medical College, Huazhong University of Science and Technology Wuhan China; ^3^ Alloimmunity, Autoimmunity and Transplantation Université de Paris, Institut de Recherche Saint‐Louis, EMiLy, INSERM U1160 Paris France; ^4^ Department of Immunology Institute of Biomedical Sciences University of São Paulo (USP) São Paulo São Paulo Brazil; ^5^ Coxiella Pathogenesis Section, Laboratory of Bacteriology Rocky Mountain Laboratories National Institute of Allergy and Infectious Diseases, National Institutes of Health Hamilton Montana USA; ^6^ Division of Molecular Pathology Research Institute for Biomedical Sciences (RIBS) Tokyo University of Science Noda Chiba Japan; ^7^ Department of Ophthalmology Rigshospitalet Hospital Copenhagen University Copenhagen Denmark; ^8^ Department of Paediatrics and Adolescent Medicine Li Ka Shing Faculty of Medicine The University of Hong Kong Hong Kong China; ^9^ Department of Pathogen Biology School of Basic Medicine Tongji Medical College and State Key Laboratory for Diagnosis and treatment of Severe Zoonotic Infectious Disease, Huazhong University of Science and Technology Wuhan Hubei China; ^10^ Department of Laboratory Medicine Institute of Biomedicine, University of Gothenburg Gothenburg Sweden; ^11^ Department of Hematology The Second Hospital of Anhui Medical University Hefei China

**Keywords:** adaptive immunity, disease pathogenesis, immunotherapy, innate immunity

## Abstract

The innate immune system serves as the body's first line of defense, utilizing pattern recognition receptors like Toll‐like receptors to detect pathogens and initiate rapid response mechanisms. Following this initial response, adaptive immunity provides highly specific and sustained killing of pathogens via B cells, T cells, and antibodies. Traditionally, it has been assumed that innate immunity activates adaptive immunity; however, recent studies have revealed more complex interactions. This review provides a detailed dissection of the composition and function of the innate and adaptive immune systems, emphasizing their synergistic roles in physiological and pathological contexts, providing new insights into the link between these two forms of immunity. Precise regulation of both immune systems at the same time is more beneficial in the fight against immune‐related diseases, for example, the cGAS–STING pathway has been found to play an important role in infections and cancers. In addition, this paper summarizes the challenges and future directions in the field of immunity, including the latest single‐cell sequencing technologies, CAR‐T cell therapy, and immune checkpoint inhibitors. By summarizing these developments, this review aims to enhance our understanding of the complexity interactions between innate and adaptive immunity and provides new perspectives in understanding the immune system.

## Graphical abstract

This review begins with a complete description of the composition and function of innate and acquired immunity. On this basis we summarize how the two systems interact and influence disease progression. The paper concludes with a review of therapeutic options and future directions for immune system research.

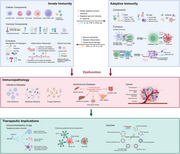

## 1 INTRODUCTION

The immune system comprises a complex network involving organs, leukocytes, proteins, and various chemicals. These components work together to generate a protective response against pathogens (such as bacteria, viruses, and parasites) and abnormal cells (such as tumors and transplanted cells), while recognizing the host organism and limiting damage to itself. The interplay between the innate and adaptive immune systems is required for complete immune function. The innate immune system is formed when the organism is born and does not undergo specific transformation according to the characteristics of the pathogen during infection. In contrast, the central feature of adaptive immunity is the ability of specialized immune cells, mainly T and B cells, to undergo genetic reorganization in response to specific antigens.^1^

In 1989, Charles Janeway Jr. first proposed the paradigm of innate immunity controlling adaptive immunity,^2^ providing a solid foundation for subsequent research. The innate immune system serves as the first line of defense following pathogen invasion, primarily relying on mucosal barriers, innate immune cells such as macrophages, mast cells, neutrophils, and basophils, along with cytokines to eliminate foreign entities. Subsequent studies have refined this basic theory, summarizing four mechanisms by which innate immunity triggers an adaptive response. The first signal is initiated by antigenic peptides on the major histocompatibility complex (MHC) recognized by the T/B cell receptor (TCR/BCR). The second one is composed of immune checkpoint (IC) molecular pairs. Cytokines are the third type of signaling. The last type is more recently discovered and its main idea is that metabolism‐associated danger signals (MADSs) are recognized by metabolic sensors (MSs).^3^, ^4^, ^5^ Although some studies have found that adaptive immunity can be independent of innate immunity, such as type II immune responses triggered by allergens and parasites,^6^ innate immunity still plays a major role in activating adaptive immunity.

As immunity has been studied in depth, researchers have discovered that the way the two immune systems interact is far more complex than imagined. Due to their close linkage, abnormalities in the functioning of one system will inevitably cause dysfunction in the other. For example, overexpression of the stimulator of interferon genes (STING) gene, which is traditionally thought to play a role in the innate immune system, can stimulate the function of cytotoxic T cells, leading to a decrease in the number of CD4+ and CD8+ T cells.^7^, ^8^ Therefore, when developing drugs to modulate the function of the immune system, the effects on both immune systems need to be fully considered. For example, although adaptive immunity plays a dominant role in tumor pathogenesis, effective activation of innate immunity can also indirectly strengthen adaptive immunity to inhibit cancer development.

To provide an in‐depth summary of the ways in which the two immune systems interact, this review systematically describes the composition and function of innate and adaptive immunity. The focus is on the crosstalk between the two immune systems and how they function together in diseases including infections, autoimmune diseases and cancer. Finally, we summarize the current research breakthroughs and future research directions in the field of immunity.

## 2 INNATE IMMUNE SYSTEM

The innate immune response represents the first line of defense against pathogens within the body. Consequently, we outline the components and the key functions of the innate immune system to enhance understanding.

### 2.1 Components of the innate immune system

The innate immune system is composed of barrier structures, effector molecules, and innate immune cells. Here, we briefly present the composition and main functions of each part.

#### 2.1.1 Barrier structures

The innate immune barrier structures are the most immediate form of immune defense in the body, which comprise physical, chemical, and microbial barriers. Physical barriers are characterized by multiple layers of squamous epithelial cells and mucosal epithelial cells on the surface of the skin, forming a protective barrier through tight junctions to prevent the invasion of pathogens.^9^ The mucosa includes the gastrointestinal tract, respiratory tract, eye, nose, mouth, urogenital tract, and so on. Chemical barriers refer to secretions produced by the appendages of the skin and mucosa, such as sebum, saliva, and tears, which contain various bactericidal and bacteriostatic substances.^10^, ^11^ Moreover, microbial barriers consist of commensal bacteria residing on the surface of the skin and mucosa.^12^, ^13^ They limit the growth and colonization of pathogens through nutrient competition and ecological niche occupation. Commensal bacteria also produce antimicrobial substances, such as lactic acid produced by lactobacilli and antimicrobial peptides (AMPs) produced by certain bacteria, which enhance the host's defense capabilities.^14^ Furthermore, the intestinal microbiome has been reported to have complex interactions with the innate immune system.^15^

#### 2.1.2 Cellular components

Innate immune cells are widely distributed in different tissues and organs of the body and primarily include all cells involved in innate and adaptive immune responses except αβT cells and B2 cells. Among them, dendritic cells (DCs), granulocytes, monocytes and macrophages belong to mononuclear phagocytes.

##### 2.1.2.1 Dendritic cells

DCs, named for their mature cells bearing numerous dendritic or pseudopodial processes, can differentiate from myeloid progenitors and lymphoid progenitors in the bone marrow, termed myeloid DCs, lymphoid DCs (or plasmacytoid DCs, pDCs), respectively. Both types of DCs migrate from the bone marrow into peripheral blood and then redistribute to various tissues throughout the body.^16^, ^17^ DCs are widely distributed in all tissues and organs except the brain, and located in different anatomical sites and at various stages of differentiation exhibit distinct nomenclature, phenotypes, and biological features. This widespread distribution is critical to their role in surveilling for pathogens and initiating an immune response.^14^, ^18^

The primary function of DCs, well known as antigen‐presenting cells (APCs), is antigen uptake, processing, and presentation to induce immune responses. Immature DCs are capable of capturing antigens through a number of different mechanisms, including micropinocytosis, phagocytosis, and receptor‐mediated endocytosis. Antigens taken up by DCs undergo processing and are presented to T cells in the form of peptide–MHC I complexes (pMHC), thereby activating naïve T cells. Additionally, DCs secrete various cytokines and chemokines, participating in the regulation of immune cell differentiation, development, activation, and effector functions, thereby influencing the direction, efficacy, and outcome of immune responses. Furthermore, DCs are involved in inducing central and peripheral immune tolerance. In the thymus, DCs participate in negative selection of T cells by eliminating self‐reactive T cells, contributing to central tolerance in T cell development. The major players in inducing peripheral immune tolerance are immature DCs, which do not express the costimulatory molecules required for T cell activation, thus inducing T cell anergy and promoting immune tolerance to self‐antigens. Moreover, immature DCs can induce regulatory T cells (Tregs) and secrete inhibitory cytokines such as IL‐10 and transforming growth factor (TGF)‐β, suppressing the activation of immune‐reactive T cells and facilitating the formation of peripheral tolerance.^19^

##### 2.1.2.2 Granulocytes

Arising from hematopoietic stem cells in the bone marrow, granulocytes undergo differentiation and development within the bone marrow before entering the bloodstream. Granulocytes possess abundant lysosomes in their cytoplasm, which are referred to as granules due to their appearance under microscopy. These granules primarily contain proteinases and other degradative enzymes and, owing to their differential staining properties, can be categorized into neutrophils, eosinophils, and basophils.

Neutrophils, as the largest subset of granulocytes, uniformly distribute numerous granular components in their cytoplasm, including various acid hydrolases, peroxidases, and lysozymes, capable of digesting engulfed bacteria and foreign particles. Additionally, their secretory granules contain lysozyme and defensins, exhibiting bactericidal properties. Neutrophils stand at the forefront of the body's defense against pathogenic microorganisms. During infection, neutrophils, guided by adhesion molecules and chemotactic factors, exit the bloodstream and vasculature through a multistep process to become the first immune cells to arrive at sites of inflammation. Apart from their role in combating infection, neutrophils can exert antibody‐dependent cell‐mediated cytotoxicity (ADCC) by binding to the Fc portion of IgG molecules on the surface of target cells via their Fc receptors. They can also phagocytose immune complexes via Fc and complement receptors, degranulating during this process and releasing an array of lysosomal enzymes, thereby causing vascular and tissue damage. Neutrophils also participate in pathological damage induced by rapid hypersensitivity reactions. Furthermore, they regulate excessive inflammatory responses by releasing anti‐inflammatory cytokines.^19^, ^20^

Eosinophils are characterized by large, closely packed eosinophilic granules in their cytoplasm, containing various enzyme components such as acid phosphatases, peroxidases, and histaminase. The primary function of eosinophils is defense against parasites such as worms and helminths.^21^ Eosinophils adhere to parasite surfaces via Fc and complement receptors, releasing granule contents to kill parasites. They also possess phagocytic capability, engulfing small pathogens or IgE‐containing immune complexes, with lysosomes in the cytoplasm capable of enzymatic digestion. Additionally, eosinophils play a role in inflammation by secreting cytokines. Basophils contain irregularly shaped and variably sized basophilic granules in their cytoplasm, which contain substances such as histamine, heparin, and proteolytic enzymes. Mast cells, found in mucosal and connective tissues, are large granule‐bearing cells in the cytoplasm with functions closely resembling those of basophils. Both basophils and mast cells express IgE receptors on their surface, undergoing degranulation and releasing inflammatory mediators upon IgE antibody action, thereby playing crucial roles in allergic immune responses.^22^, ^23^, ^24^, ^25^

##### 2.1.2.3 Monocytes and macrophages

The mononuclear phagocyte system (MPS) comprises premonocytes, monocytes, and tissue macrophages. The MPS originates from hematopoietic stem cells in the bone marrow. Under the influence of certain cytokines such as macrophage colony‐stimulating factor (M‐CSF) and monocyte growth factor, hematopoietic stem cells develop into premonocytes, which further differentiate into monocytes and enter the bloodstream. Monocytes remain in the circulation for several hours to days before traversing endothelial cells and entering various tissues and organs throughout the body, where they mature into macrophages. Macrophages are predominantly tissue‐resident and can be found in diverse environments such as the liver (Kupffer cells), brain (microglia), and lungs (alveolar macrophages). The distribution of macrophages is crucial for their role in maintaining tissue homeostasis and responding to pathologic conditions. Mature monocytes and macrophages express a variety of surface molecules, including Fc receptors, complement receptors, and various pattern recognition receptors (PRRs) such as mannose receptor, scavenger receptor (SR), and Toll‐like receptors (TLR). Activated monocytes and macrophages also express MHC class I and II molecules associated with antigen presentation, chemokine receptors, and adhesion molecules related to chemotaxis and adhesion. Moreover, they secrete various cytokines, small‐molecule inflammatory mediators, and complement components, participating in inflammation and immune regulation.^26^ Macrophages are further characterized by their different functional phenotypes. They are usually divided into two groups according to their activation mode, which are classically activated type I macrophages (M1) and alternative activated type II macrophages (M2). There are notable differences in the surface receptor expression, cytokine and chemokine production, effector functions, and intracellular signaling pathways exhibited by M1 and M2 macrophages, contingent on the activation mode.^27^ Macrophages can not only phagocytose, digest, and eliminate large particle antigens such as pathogens, exerting an anti‐infective role, but also engulf and clear senescent, dying, or transformed cells, thereby maintaining immune homeostasis. Additionally, macrophages, as APCs, can uptake, process, and present antigens by presenting antigen pMHC class I complexes to CD4+ T cells.

##### 2.1.2.4 Natural killer cells

Natural killer (NK) cells differentiate from lymphoid progenitor cells in the bone marrow and belong to the lymphoid lineage, expressing various lymphocyte markers. However, NK cells do not express antigen‐specific receptors such as TCR and BCR, and morphologically differ from lymphocytes. They migrate to various tissues throughout the body after differentiating from hematopoietic stem cells. They are primarily distributed in peripheral blood and are also present in tissues such as the liver, spleen, lungs, and lymph nodes.^28^ NK cells are named for their ability to target and kill virus‐infected cells and malignant cells without prior sensitization. They can rapidly activate through binding to specific antigens on the surface of target cells after binding to IgG antibodies via surface IgG Fc receptors (CD16), thereby mediating ADCC to kill target cells. NK cells can also induce target cell lysis by releasing perforin and granzymes or induce target cell apoptosis via the Fas/FasL pathway. Through various regulatory receptors such as activating receptors and inhibitory receptors, NK cells selectively kill abnormal or diseased cells without harming normal tissue.^29^ Furthermore, NK cells can influence other types of cells such as DCs, T cells, B cells, and endothelial cells through cell‐cell interactions and cytokine secretion, thereby exerting immunomodulatory effects.^28^

##### 2.1.2.5 Innate‐like lymphocyte

There are lymphocytes that, although sharing a common cellular origin with T cells and B cells, have very limited diversity in antigen receptors and do not involve clonal selection and expansion for antigen recognition and activation. These lymphocytes include NK T (NKT) cells, γδT cells, B1 cells, marginal zone (MZ) B cells, and innate lymphoid cells (ILCs).

NKT cells are a subset of T cells that express both TCR and certain NK cell surface markers, which have limited TCR diversity and a narrow antigen recognition spectrum. NKT cells mainly reside in the liver and bone marrow. Upon activation, NKT cells can rapidly secrete a large amount of Th1 and Th2 cytokines, thereby exerting immunomodulatory effects. Additionally, NKT cells can promote cell‐mediated immunity against tumors and infectious agents and are also associated with the development of autoimmune diseases.^30^, ^31^, ^32^ Another type of innate‐like T cells (ILTs) is γδT cells. Unlike αβT cells, which are the important component of adaptive immunity, the TCR of these T cells consists of γ and δ chains. They are predominantly CD4^−^CD8^−^ cells and constitute only 1–10% of the total CD3^+^ T cell population.^33^, ^34^ γδT cells are distributed in mucosal and subcutaneous tissues such as the skin, small intestine, lungs, central nervous system, and reproductive organs, being part of the intraepithelial lymphocytes. γδT cells mainly recognize unprocessed peptide antigens and certain nonpeptide antigens presented by CD1, rather than antigen pMHC complexes. γδT cells are important components of nonspecific immune defense, particularly playing crucial roles in local mucosal immunity and hepatic immune responses to infections, as well as in immune surveillance and homeostasis.^33^, ^34^, ^35^

Based on the sequence of appearance during fetal development, B cells can be divided into three classes, which are B1 cells, B2 cells, and MZ B cells. B2 cells, commonly referred to as B cells participating in adaptive humoral immune responses, are distinct from B1 cells and MZ B cells, which are collectively referred to as innate‐like B cells.^36^, ^37^ B1 cells are mainly distributed in the pleural and peritoneal cavities and the lamina propria of the intestine, with a narrow antigen recognition spectrum, primarily recognizing polysaccharide TI‐2 antigens, especially certain polysaccharide antigens shared by bacterial surfaces. B1 cells mainly produce low‐affinity IgM antibodies, do not undergo class switching, and lack immunological memory.^38^, ^39^, ^40^ In contrast to B1 cells, MZ B cells primarily reside in the splenic red pulp and MZ, secreting IgM to participate in immune responses. Additionally, MZ B cells can influence the function of T cells and DCs through cytokine production.^41^

ILCs are a type of lymphocytes lacking TCR and BCR, mainly including ILC1, ILC2, and ILC3 subgroups, with NK cells and lymphoid tissue inducer cells broadly classified as ILCs.^42^ ILCs are considered innate counterparts of effector CD4+ T cells, where ILC1 functions similarly to Th1, ILC2 to Th2, and ILC3 to Th17. ILCs are typically found in lymphoid tissues and peripheral organs, especially in the skin, liver, small intestine, and lungs. They play essential roles in inflammation activation, tissue remodeling, metabolic control, and influencing adaptive immune responses.^1^, ^43^, ^44^

#### 2.1.3 Humoral components

Among the various effector molecules involved in immune responses and inflammatory reactions, with the exception of antibodies, which are adaptive immune effector molecules, the rest are involved in innate immunity. These include complement, cytokines, lysozyme, AMPs, and so on. This part mainly focuses on complement and cytokines.

##### 2.1.3.1 The complement system

The complement system is a crucial component of the innate immune response, playing significant roles in combating infections, clearing immune complexes, and regulating inflammation. It consists of a series of small proteins circulating in the blood in an inactive form under normal conditions. Upon activation, these proteins undergo a cascade of proteolytic cleavages, resulting in various immune responses.

Complement activation can be divided into two phases, and their dividing line is the formation and activation of C3 convertase. The activation of C3 convertase occurs via three pathways, which are the classical pathway, the lectin pathway, and the alternative pathway.^45^, ^46^ The classical pathway is initiated when antibodies, mainly IgM and IgG, recognize and bind to antigens on the surface of pathogens or cells, forming immune complexes. Antibodies bound to antigens can then bind C1q, sequentially activating C1r and C1s to form the C1 complex. Activated C1s cleaves C4 and C2 to form the C4b2b complex, which is C3 convertase. The lectin pathway is initiated by mannose‐binding lectin (MBL) or ficolins, which selectively recognize carbohydrate structures on various pathogens, including mannose, fucose, and N‐acetylglucosamine. Upon recognition of pathogen‐associated carbohydrate chains, MBL‐associated serine proteases are activated, which mimic C1s activity, and then activate the C3 convertase, initiating the complement cascade. Unlike the classical and lectin pathways, the alternative pathway can directly activate C3, bypassing C4 and C2. Under physiological conditions, serum C3 undergoes slow and continuous hydrolysis, producing low levels of C3b. Once pathogens enter the body, they can be adhered to C3b, and with the participation of serum factors B, D, and P, the C3 convertase is formed. C3 convertases in all three pathways cleave the substrate C3 into C3a and C3b. In conclusion, the initiation of the classical pathway depends on the antigen–antibody complexes, and thus complement participates in part of the adaptive immune response. But the other two pathways do not involve antibodies, suggesting that complement plays an important role in the natural defense system.^47^, ^48^

Following the formation and activation of C3 convertase, subsequent cascade reactions, including the formation of C5 convertase, can exert cytolytic effects, mediate inflammation, opsonize pathogens, and clear immune complexes.^46^, ^49^, ^50^ The C3b generated by C3 convertase can combine with C4b2b or C3bBb to form C5 convertase (C4b2b3b or C3bBb3b). C5 convertase cleaves C5 into C5a and C5b. C5b can bind to the cell membrane and sequentially recruit C6, C7, and C8 to form the C5b678 complex, which integrates into the cell membrane. This complex can then bind multiple C9 molecules to form the membrane attack complex (MAC, C5b6789n). MAC forms transmembrane channels approximately 11 nm in diameter, allowing the free flow of water, ions, and small molecules. The formation of numerous MACs on the target cell surface leads to osmotic imbalance, causing the cell to swell and eventually lyse. If MAC formation results from the classical pathway, the consequent cell death is termed complement‐dependent cytotoxicity. Additionally, small fragments generated during complement activation, such as C4a, C3a, and C5a, act as anaphylatoxins, inducing local inflammation and recruiting phagocytes. C3b covalently binds to pathogen surfaces, where phagocytes, which possess receptors recognizing C3b, enhance the phagocytosis and clearance of C3b‐opsonized pathogens. This opsonization function of complement is a major mechanism in the defense against systemic bacterial and fungal infections. Last, complement components participate in clearing circulating immune complexes. Mechanistically, C3b covalently binds to immune complexes, adhering to erythrocytes and platelets, which transport immune complexes to the liver and spleen for macrophage‐mediated clearance. C3b can also bind to immunoglobulins (Igs), reducing the affinity between antibody Fab fragments and antigens, thereby inhibiting immune complex formation. C3b can dissociate preformed immune complexes by integrating into their lattice structure as well.

##### 2.1.3.2 Cytokines

Cytokines are small proteins that play crucial roles in cell signaling, particularly within the immune system. They are secreted by a variety of cells, such as macrophages, B and T lymphocytes, mast cells, endothelial cells, and fibroblasts. Cytokines can be broadly categorized into several types, including interleukins (ILs), interferons, tumor necrosis factors (TNFs), CSFs, and chemokines. Each type performs specific functions in the immune response (Table 1). For instance, ILs are primarily involved in communication between leukocytes, interferons are critical for antiviral defense, TNFs are involved in systemic inflammation as part of the acute phase reaction, CSFs stimulate the production of blood cells, and chemokines induce chemotaxis of nearby responsive cells. For detailed functions, we recommend consulting the review article on this topic.^14^, ^51^

**TABLE 1:** Basic classification of cytokines.^52^

Cytokine type	Main members		Functions
Interleukins	From IL‐1 to IL‐35		Widely involved in immune regulation, hematopoiesis, and inflammatory processes
Chemokines	CXC	IL‐8, GRO, PBP, IP‐10, SDF‐1, and PF‐4	Primarily chemotactic for neutrophils
	CC	MIP‐1α, MIP‐1β, RANTES, MCP‐1, MCP‐2, and MCP‐3	Primarily chemotactic for monocytes
	CX3C	Fractalkine	Chemotactic for NK cells, T cells, and macrophages
	C	LTN and SCM‐1β	Chemotactic for T cells and bone marrow cells
Colony‐stimulating factors	G‐CSF, M‐CSF, GM‐CSF, IL‐3, SCF, EPO, and TPO		Stimulate proliferation and differentiation of hematopoietic stem and progenitor cells at various developmental stages and enhance the function of mature cells
Interferons	Type I	IFN‐α, IFN‐β, IFN‐ε, IFN‐κ, and IFN‐ω	Involved in antiviral, antiproliferative, antitumor immunity, and immunomodulatory
	Type II	IFN‐γ	
	Type III	IFN‐λ	
Tumor necrosis factors	TNF‐α, TNF‐β (LT‐α), and LT‐β		Involved in killing target cells, immune regulation, inflammatory responses, and induction of apoptosis
Growth factors	TGF, EGF, VEGF, and FGF		Promote growth and differentiation of respective cells

Abbreviations: EGF, epidermal growth factor; EPO, erythropoietin; FGF, fibroblast growth factor; G‐CSF, granulocyte colony‐stimulating factor; GM‐CSF, granulocyte‐macrophage colony‐stimulating factor; GRO, growth‐regulated oncogene; IP‐10, interferon‐inducible protein 10; LTN, lymphotactin; MCP, monocyte chemoattractant protein; M‐CSF, macrophage colony‐stimulating factor; MIP‐1, macrophage inflammatory protein 1; PBP, platelet basic protein; PF‐4, platelet factor 4; RANTES, regulated on activation, normal T cell expressed and secreted; SCF, stem cell factor; SDF‐1, stromal cell‐derived factor 1; and TGF, transforming growth factor; TPO, thrombopoietin; VEGF, vascular endothelial growth factor.

The biological actions of cytokines are highly complex, as different cytokines can act on the same type of cell, producing identical or similar biological effects. Moreover, the same cytokine can exhibit completely opposite effects in different microenvironments or on different target cell types. The effects of various cytokines are interrelated, as their synthesis, secretion, and receptor expression can mutually regulate each other, forming a highly complex cytokine network that performs essential biological functions. Cytokines are indispensable in the regulation of immune and inflammatory responses. They can promote or inhibit the proliferation and differentiation of various cell types, modulate the balance between humoral and cell‐based immune responses, and orchestrate the migration of cells to sites of infection or injury. Dysregulation of cytokine production or signaling is implicated in numerous pathological conditions, including autoimmune diseases, chronic inflammatory diseases, and cancer.^14^

### 2.2 Functions of the innate immune system

The innate immune system serves as a rapid and broad‐spectrum defense mechanism. This section delves into the primary functions of the innate immune system, highlighting how it recognizes pathogens, initiates inflammatory responses, and adapts through trained immunity.

#### 2.2.1 Recognition of pathogens

Innate immunity constitutes the first line of defense against pathogenic invasion, primarily mediated through PRRs that identify pathogen‐associated molecular patterns (PAMPs) and damage‐associated molecular patterns (DAMPs). PAMPs, which are highly conserved molecular structures found in bacteria, viruses, fungi, and parasites, include bacterial lipopolysaccharides (LPS), viral RNA, and fungal cell wall components, encompassing proteins, lipids, carbohydrates, and nucleotides. DAMPs are endogenous molecules released from damaged or dying cells, signaling tissue damage and promoting immune responses, These include intracellular molecules such as heat shock proteins, high‐mobility group box 1, ATP, DNA, and RNA, released upon cell damage or stress, as well as extracellular matrix components like collagen, elastin, and matrix metalloproteinases exposed during tissue injury.^14^, ^53^, ^54^

PRRs, which mediate the recognition of these molecular patterns, exhibit limited diversity and nonclonal expression, Different PRRs recognize common molecular patterns from various pathogens, enabling a limited number of PRRs to detect a wide array of PAMPs and DAMPs without antigen specificity. PRRs can be located on the cell membrane, within the cytoplasm, or in bodily fluids and are categorized into four main types based on their functions (Table 2). The first type is secreted PRRs, such as MBL, which can activate the complement system through the MBL pathway or mediate opsonization, enhancing pathogen clearance. C‐reactive protein (CRP) is another example, binding to phosphocholine on bacterial cell walls to facilitate opsonization or complement activation. The second type includes membrane‐bound phagocytic receptors, which recognize and bind PAMPs, internalizing pathogens into cytoplasmic vesicles for direct digestion and clearance. Key examples are C‐type lectin receptors (CLRs) like the mannose receptor, which specifically binds terminal mannose and fucose residues in microbial cell wall glycoproteins and glycolipids, mediating phagocytosis by macrophages. SRs also fall into this category, binding to various bacterial cell wall components and efficiently clearing bacteria from the bloodstream. The third type comprises membrane‐bound signaling receptors, exemplified by TLRs. Over 10 TLRs have been identified in mammals, primarily located on the cell membrane, with some, such as TLR3, TLR7, TLR8, TLR9, and TLR13, found on endosomal membranes.^55^ TLRs recognize diverse microbial molecules such as LPS, peptidoglycan, and viral nucleic acids, initiating signaling cascades that lead to the production of proinflammatory cytokines and interferons.^56^, ^57^ The fourth type encompasses cytoplasmic signaling receptors, including NOD‐like receptors (NLRs), retinoic acid‐inducible gene‐I (RIG‐I)‐like receptors (RLRs), and cytosolic DNA sensors (CDS). NLRs include subfamilies such as NLRC and NLRP, which detect bacterial cell wall peptidoglycans, LPS, uric acid crystals, and damaged cell products, triggering intracellular signaling pathways that often activate inflammasomes. Inflammasomes are multiprotein complexes composed of NLRs, adaptor proteins like ASC, and effector molecules such as caspase‐1. They recognize both exogenous PAMPs and endogenous DAMPs, directly activating caspase‐1, which in turn processes IL‐1β and IL‐18 into their active forms and induces pyroptosis, a form of programmed cell death.^58^ Furthermore, RLRs detect viral RNA, leading to the production of type I interferons and other antiviral responses. Unlike RLRs, CDSs specifically recognize viral and bacterial DNA, engaging signaling pathways involving molecules such as STING, DNA‐dependent activator of interferon‐regulatory factors, and absent in melanoma 2 (AIM2). For instance, STING, a transmembrane protein on the endoplasmic reticulum, is activated by cyclic GMP–AMP synthase (cGAS) upon binding to cytosolic dsDNA. This activation induces conformational changes, translocating STING to function as a platform for recruiting and activating TANK‐binding kinase 1 (TBK1), which subsequently phosphorylates interferon regulatory factor 3 (IRF3). Phosphorylated IRF3 then translocates to the nucleus to promote the expression of type I interferon genes, producing interferons essential for antiviral responses.

**TABLE 2:** Basic classification of pattern recognition receptors.^14^, ^59^

PRR type	Main members	Examples	Main legends	Functions
Secreted receptors	Collectin	MBL	Oligosaccharides rich in mannose	‐ Opsonization functions ‐ Complement activation
	Pentraxin	CRP	Phosphatidylcholine	‐ Opsonization functions ‐ Complement activation
Membrane‐bound phagocytic receptors	CLR	MR	Oligosaccharides rich in mannose	‐ Phagocytosis
	SR	SR‐A and SR‐B	Diacylglycerol	‐ Phagocytosis ‐ Involved in lipoprotein metabolism
Membrane‐bound signaling receptors	TLR	TLR1, TLR2, TLR4, TLR5, TLR6, TLR11 on cell surface. TLR3, TLR7, TLR8, TLR9, and TLR13 on endosomal membrane	Various microbial molecules such as LPS, peptidoglycan, and viral nucleic acids	‐ Activation of intracellular signaling (mainly MyD88 signaling) ‐ Induction of adhesion molecules and inflammatory cytokines
Cytoplasmic signaling receptors	NLR	NOD1, NOD2, and NLRP3	Bacterial cell wall peptidoglycan, flagellin, cell wall peptidoglycan, LPS, uric acid crystals, damaged cell products	‐ Activation of intracellular signaling ‐ Activation of inflammasomes ‐ Induction of inflammatory cytokines
	RLR	RIG‐1, MDA‐5	Viral RNA	‐ Activation of intracellular signaling ‐ Induction of interferon production
	CDS	STING, AIM2	Bacterial and viral DNA	‐ Activation of intracellular signaling ‐ Induction of interferon production

#### 2.2.2 Initiation of inflammatory responses

Innate immunity initiates an inflammatory response to combat infection and facilitate tissue repair. This response is mediated by cytokines, chemokines, plasma enzyme mediators, and lipid inflammatory mediators produced by immune cells upon recognizing pathogens or tissue damage. Cytokines, such as ILs, TNFs, and interferons, induce local inflammatory responses, including endothelial cell and lymphocyte activation and increased vascular permeability, and can trigger systemic effects like fever. Chemokines specifically attract responsive cells to the site of infection or injury, directing immune cells to the affected area. Four plasma enzyme systems, which are the kinin, coagulation, fibrinolytic, and complement systems, are activated upon tissue damage, generating a plethora of inflammatory mediators. Additionally, inflammatory cells such as macrophages, neutrophils, and mast cells degrade membrane phospholipids into arachidonic acid and platelet‐activating factors (PAFs). Arachidonic acid is converted via the cyclooxygenase pathway into prostaglandins (PGE) and thromboxanes. PGE increases vascular permeability and induces vasodilation, while thromboxanes promote platelet aggregation and vasoconstriction. The lipoxygenase pathway also transforms arachidonic acid into leukotrienes, which are potent mediators produced by mast cells. PAFs not only activate platelets but also induce degranulation of neutrophils and eosinophils.^60^, ^61^

Inflammatory responses can be classified into acute and chronic phases. Acute inflammation is the immediate response to short‐term, localized infection or injury, characterized by redness, swelling, heat, pain, and functional impairment. These symptoms result from vasodilation, increased vascular permeability, leukocyte infiltration, and the release of inflammatory mediators. Chronic inflammation arises from the persistent presence of antigens and denotes a prolonged inflammatory process, typically occurring when an infection is not completely eradicated or recurrently occurs. This phase is characterized by chronic inflammatory cell infiltration, tissue fibrosis, and functional impairment. During chronic inflammation, cells like lymphocytes, monocytes, and macrophages accumulate at the site, forming a chronic inflammatory infiltrate. Chronic inflammation also involves tissue remodeling and fibrosis, marked by fibroblast activation and collagen deposition, leading to altered tissue structure and dysfunction.^61^, ^62^

#### 2.2.3 Trained immunity

Upon infection, cells involved in innate immunity can establish and maintain long‐term functional changes. This phenomenon, designated as trained immunity, is distinguished by an augmented production of inflammatory mediators and an augmented capacity to eliminate pathogens upon subsequent encounters. In contrast to adaptive immune memory, trained immunity is not dependent on lymphocytes and does not necessitate antigen specificity, thereby enabling it to respond to a range of pathogens.^63^

The mechanisms underlying trained immunity involve metabolic reprogramming, epigenetic modifications, and the production of inflammatory mediators. Following pathogen exposure, innate immune cells such as macrophages and DCs undergo metabolic shifts, including increased glycolysis and fatty acid oxidation, to meet heightened energy demands. Concurrent with these metabolic changes, these cells exhibit alterations in epigenetic marks, such as histone methylation and acetylation, which influence gene expression. Additionally, innate immune cells produce various inflammatory cytokines and chemokines, such as TNF, IL‐1β, and IL‐6, which play crucial roles in the infection response and may contribute to the establishment of trained immunity.^63^, ^64^

## 3 ADAPTIVE IMMUNE SYSTEM

In this section, we will provide a detailed overview of the adaptive immune system and its components, focusing on the key role of T and B cells in the immune response. We will describe antigen recognition and cellular immunity of T cells, humoral immunity of B cells, and how the adaptive immune system generates immune memory to safeguard the body's long‐term immune defenses.

### 3.1 Components of the adaptive immune system

#### 3.1.1 T cells

Lymphocytes are a primary cell type of the immune system, predominantly found in lymphoid organs, lymphoid tissues, and peripheral blood. The cells involved in adaptive immune responses are T and B lymphocytes. T lymphocytes, or T cells, are crucial for defending the body against pathogens such as viruses and bacteria, as well as for monitoring and eliminating cancer cells. T cells are characterized by their surface TCRs, which enable them to recognize specific antigens presented by other cells, a recognition critical for initiating and coordinating immune responses.

T cells originate in the bone marrow and mature in the thymus. Within the thymus, T cells undergo several developmental stages, including the critical phases of positive and negative selection. Positive selection ensures the survival of T cells with functional TCRs capable of interacting with MHC molecules, while negative selection eliminates T cells that strongly react to self‐antigens. Surviving mature T cells differentiate into various subsets, each with specific functions in immune responses. Helper T cells (CD4+ T cells) secrete cytokines to activate and direct other immune cells, playing a key role in coordinating immune responses. They further differentiate into subgroups such as Th1, Th2, and Th17, each with distinct roles in immune regulation and response. Cytotoxic T cells (CD8+ T cells) recognize antigens presented by MHC class I molecules and directly kill infected or cancerous cells. Tregs are essential for maintaining immune tolerance and preventing autoimmune diseases by suppressing excessive immune responses. Memory T (TM) cells form after initial antigen exposure and provide a rapid and robust response upon subsequent encounters with the same antigen, thus ensuring long‐lasting immunity.

One of the main functions of T cells is cytokine production. For instance, Th1 cells produce interferon‐gamma (IFN‐γ), which activates macrophages and enhances their ability to phagocytose pathogens. Th2 cells release IL‐4, which stimulates B cells to produce antibodies. Th17 cells secrete IL‐17, which is involved in recruiting neutrophils to infection sites. Regulatory Tregs produce inhibitory cytokines such as IL‐10 and TGF‐β, helping to suppress excessive immune responses^65^ and maintain immune tolerance. Direct cytotoxicity is another key function primarily executed by cytotoxic T cells. These cells recognize and bind to infected or cancerous cells presenting antigens via MHC class I molecules and kill the target cells by secreting perforin and granzymes or inducing apoptosis through the FasL/Fas pathway. Additionally, they can exert cytotoxic effects by secreting cytokines such as TNF‐α.^14^, ^19^

#### 3.1.2 B cells

B lymphocytes, or B cells, are primarily responsible for humoral immunity. B cells recognize specific antigens through BCRs, leading to their activation and subsequent production of antibodies to neutralize or eliminate the recognized pathogens. Beyond antibody production, B cells also function as APCs and secrete cytokines that regulate immune responses. The significance of B cells in the immune system is profound, as they contribute not only to immediate defense mechanisms but also play a crucial role in long‐term immunity through the formation of memory B cells (MBCs).

B cell development occurs in two stages, which are central development and peripheral development. Central development involves the differentiation and maturation of progenitor B cells from hematopoietic stem cells in the bone marrow. This process begins with the rearrangement of Ig genes, known as V(D)J recombination, which is essential for generating a diverse repertoire of BCRs capable of recognizing various antigens. Once B cells express functional BCRs, they undergo negative selection, where immature B cells that strongly bind to self‐antigens are eliminated to prevent autoimmunity. Mature B cells express surface markers such as mIgM, mIgD, CD19, CD21, and CD81, as well as receptors for complement, mitogens, and cytokines. These mature B cells enter the bloodstream and migrate to peripheral lymphoid organs, such as the spleen and lymph nodes, where they remain as naïve B cells until they encounter antigens. Upon antigen stimulation, B cells proliferate and differentiate into plasma cells (PCs), producing antibodies in a process known as peripheral development. When naïve B cells encounter their specific antigens and receive additional signals from helper T cells, they can differentiate into several types of cells with distinct functions. PCs, the effector cells of B cells, produce and secrete large quantities of specific antibodies against the encountered antigens. MBCs, which persist in the body after the initial infection is cleared, provide a rapid and robust antibody response upon re‐exposure to the same antigen. This developmental and differentiation process ensures that B cells play a diverse and critical role in maintaining immune defense and homeostasis.^14^, ^19^

#### 3.1.3 Antibodies

Antibodies, also known as Igs, are effector molecules produced by PCs following the antigen‐specific activation of B cells. They play a crucial role in mediating humoral immunity by recognizing and neutralizing foreign invaders such as bacteria, viruses, and toxins. Antibodies can be classified into two types, which are secreted antibodies and membrane antibodies. Secreted antibodies are primarily found in blood and tissue fluids, where they perform anti‐infective functions. Membrane antibodies, also known as BCRs, recognize and bind specific antigens, thereby activating B cells.

Antibodies are composed of four polypeptide chains, two heavy (H) chains and two light (L) chains, which are connected by disulfide bonds to form a Y‐shaped molecule with a symmetric structure. Each light chain, with a molecular weight of approximately 25 kDa, can be either of two types, κ and λ. Heavy chains, larger at about 50 kDa each, are classified into five types, which are μ, δ, γ, α, and ε, determining the antibody isotypes IgG, IgA, IgM, IgE, and IgD, respectively. The N‐terminal region of both heavy and light chains, approximately 110 amino acid residues, shows significant variability and is termed the variable region (V), while the remaining amino acid sequences are relatively constant, known as the constant region (C). Within the V regions of the heavy and light chains (VH and VL, respectively), there are three hypervariable regions or complementarity‐determining regions (CDRs) that form the antigen‐binding site. The regions outside the CDRs, which exhibit less variability, are known as framework regions. The hinge region, rich in proline and situated between the two heavy chains, provides flexibility and allows the Y‐shaped arms to adjust their distance, facilitating antigen binding. Antibodies can be enzymatically cleaved into different fragments. Papain digestion near the hinge region produces three fragments: two identical antigen‐binding fragments (Fab) and one crystallizable fragment (Fc). The Fab fragments bind antigens but do not induce aggregation or precipitation, while the Fc fragment, devoid of antigen‐binding activity, interacts with effector molecules or cells.^54^, ^66^

Antibodies exhibit several biological functions. Primarily, they specifically recognize and bind antigens through the CDRs in their V regions. This binding can neutralize pathogens or toxins by blocking their virulent structures. Additionally, antibodies can activate the complement system. Antigen–antibody complexes trigger the classical complement pathway, leading to pathogen lysis and inflammation. Aggregates of IgG4, IgA, and IgE can also activate the alternative complement pathway. Furthermore, antibodies facilitate opsonization. The Fc region of IgG binds to Fcγ receptors (FcγR) on macrophages and neutrophils after the antibody's V region binds particulate antigens, enhancing phagocytosis and destruction of pathogens. Moreover, antibodies bound to antigens on infected or cancerous cells can recruit NK cells, which possess FcγR, leading to the release of cytotoxic molecules and the killing of target cells, a process known as ADCC. Finally, antibodies can mediate hypersensitivity reactions. The diverse immunological functions of antibodies underscore their importance in maintaining health and combating disease.^19^

### 3.2 Functions of the adaptive immune system

#### 3.2.1 T cell‐mediated cellular immunity

T cell‐mediated immune responses are critical for the defense against intracellular pathogens and cancer cells. This process begins with APCs, such as DCs, macrophages, and B cells, which capture and process antigens from pathogens. APCs process these antigens into fragments suitable for binding with MHC molecules. The antigen peptides form complexes with MHC molecules and are expressed on the APC surface for T cell recognition, a process known as antigen presentation. Different pathways present antigens to T cells based on their origin, with two primary pathways discussed here. On the one hand, endogenous antigens, synthesized by APCs or host cells themselves, include viral and tumor antigens expressed by infected cells. These antigens are processed in the cytoplasm, enter the endoplasmic reticulum, and bind to MHC class I molecules, forming stable complexes. These complexes are then transported via the Golgi apparatus to the cell surface, where they are recognized by TCRs of CD8+ T cells, leading to CD8+ T cell activation and subsequent killing of the target cells presenting specific antigens. On the other hand, exogenous antigens, captured from extracellular sources like pathogens and their products, are internalized by APCs, enclosed by the plasma membrane to form vesicles in the cytoplasm. These vesicles migrate intracellularly, acidify, and eventually fuse with lysosomes. The acidic environment and proteases within endosomes and lysosomes degrade antigens into peptides. MHC class II molecules, synthesized in the ER and transported via the Golgi, form MHC class II‐containing compartments (MIIC). MIICs fuse with endosomes or lysosomes containing exogenous antigen peptides, forming pMHCII complexes. These complexes are expressed on the APC surface via exocytosis and presented to CD4+ T cells, inducing their activation, proliferation, and differentiation into effector Th cells.^14^, ^67^

From the perspective of T cells, the maturation of naive T cells occurs in the thymus, after which they enter the bloodstream and migrate to peripheral lymphoid organs. This process of recirculation between the blood and peripheral lymphoid tissues is continuous. APCs carrying pMHC complexes enter lymphoid tissues to interact with T cells. Initially, naive T cells randomly contact APCs via surface adhesion molecules like LFA‐1/ICAM, facilitating transient, reversible, nonspecific binding. This interaction allows T cells to screen for specific antigen peptides among the numerous pMHCs on the APC surface. T cells that encounter their specific antigen transmit recognition signals through CD3 molecules, enhancing adhesion molecule affinity and stabilizing T cell–APC binding until T cells proliferate and differentiate into effector cells.

**FIGURE 1:**
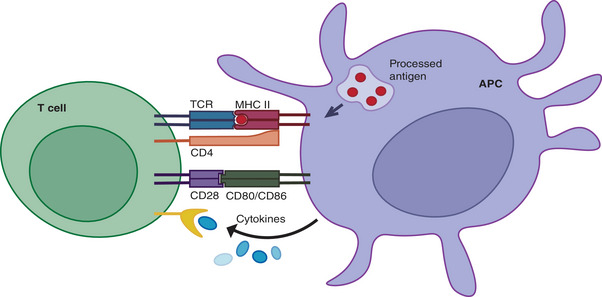
Illustration of T cell activation by APC (using CD4 T cells as an example). The TCR recognizes and binds to the pMHC complex presented by the APC. The coreceptor CD4 binds to the MHC class II molecule, generating the first signal, which induces T cell activation. Additionally, the binding of CD80 and CD86 on the APC surface to CD28 on the T cell surface provides the second signal, also known as the costimulatory signal, which further induces T cell activation and proliferation. Cytokines secreted by the APC provide the third signal to the T cells, promoting the activation, proliferation, and differentiation of T cells.

APCs provide signals that induce T cell activation, proliferation, and differentiation while presenting antigens. The first signal for T cell activation comes from the specific binding of TCRs to pMHC complexes on APC surfaces. Coreceptors, CD4 or CD8, binding to MHC molecules bring associated tyrosine kinases close to CD3 cytoplasmic domains, initiating kinase activation cascades that deliver T cell activation signals. The second signal, or costimulatory signal, arises from the binding of costimulatory molecules CD80/CD86 on APCs to CD28 on T cells, inducing T cell activation and proliferation. This costimulatory signal is essential for complete T cell activation, preventing anergy when the first signal is insufficient. Additionally, cytokines secreted by APCs provide the third signal, further promoting T cell activation, proliferation, and differentiation (Figure 1). Once activated, T cells undergo clonal expansion, proliferating, and differentiating into effector T cells. CD4+ T cells differentiate into various subpopulations, including Th1, Th2, Th17, and Tregs, each producing different cytokines and performing distinct functions in immune responses. Th1 cells produce IFN‐γ, essential for macrophage activation and intracellular pathogen clearance. Th2 cells secrete IL‐4, supporting humoral immunity by aiding B cell antibody production. Conversely, CD8+ T cells differentiate into cytotoxic T lymphocytes (CTLs), which directly kill infected or cancerous cells by releasing perforin and granzymes, inducing apoptosis in target cells. In summary, T cell activation, proliferation, and differentiation are continuous, complex processes regulated by multiple signals. The first signal determines the specificity of T cell activation, the second provides necessary activation conditions, and the third promotes T cell proliferation and differentiation. These processes are central to the immune response.^14^, ^68^, ^69^

#### 3.2.2 B cell‐mediated humoral immunity

B cell‐mediated humoral immune responses are a critical component of the immune system, primarily targeting extracellular pathogens. Antigens that elicit B cell responses can be classified into two categories: thymus‐dependent (TD) antigens and thymus‐independent (TI) antigens.

TD antigens primarily activate B2 cells. In this process, B cells function both as responders and as professional APCs. They capture, process, and present TD antigens to Th cells, facilitating their activation and helper functions. Initially, BCRs on the B cell surface recognize and bind specific epitopes on the antigen, providing the first activation signal. The B cell coreceptor complex, consisting of CD21, CD19, and CD81, also enhances this signal by augmenting BCR‐mediated antigen recognition. Once internalized, the antigen is degraded into peptides within the B cell, exposing T cell epitopes. These epitopes form complexes with MHC class II molecules, which are then transported to the B cell surface for recognition by specific Th cells. Activated Th cells express CD40L, which binds to CD40 on B cells, delivering the costimulatory second signal necessary for full B cell activation. Additionally, Th cells secrete cytokines such as IL‐4, IL‐5, and IL‐6, further promoting B cell activation, proliferation, and differentiation into PCs. B cells activated by first signal, second signal and cytokines undergo several fates. Some migrate to the medullary cords of lymphoid tissues and differentiate into short‐lived PCs that primarily secrete IgM, providing an early humoral response. Most activated B cells, however, proliferate extensively within germinal centers (GCs), undergoing clonal expansion, somatic hypermutation of antibody variable regions, affinity maturation, class switching, and receptor editing. This process culminates in the generation of high‐affinity IgG‐secreting PCs and long‐lived MBCs. PCs produce large quantities of specific antibodies that neutralize pathogens, activate the complement system, and enhance phagocytosis, thereby mediating immune defense. Upon re‐exposure to the same antigen, MBCs rapidly differentiate into PCs, thereby ensuring long‐term immunity.^14^, ^70^ TI antigens, such as bacterial polysaccharides and LPS, can directly activate resting B cells without Th cell assistance. However, TI antigens primarily stimulate B1 cells, leading to the production of low‐affinity IgM antibodies without generating immune memory. In summary, B cell‐mediated humoral immune responses involve intricate and tightly regulated interactions among various cell types and signaling molecules. Through antibody production, B cells play an indispensable role in infection defense and maintaining immune homeostasis.

#### 3.2.3 Generation of immunological memory

In the context of acquired immunity, immunological memory refers to the phenomenon wherein the host mounts a faster and more effective response upon re‐encounter with a previously sensitizing antigen. Unlike the trained memory of innate immunity, adaptive immune memory is carried out by lymphocytes, exhibiting high antigen specificity and can be categorized into cellular and humoral memory based on the mediating cells. For more understanding of adaptive immunity, we recommend this review.^71^

T cell‐mediated immune memory exhibits long‐lasting effects. As previously described, T cells undergo clonal expansion and differentiate into effector cells under antigen selection. While most effector cells undergo programmed cell death postfunction, a small fraction differentiates into TM cells. TM may also arise directly from antigen‐stimulated naïve T cells. TM cells encompass three major subsets characterized by distinct surface markers, migration, residency, and functional properties, which are effector TM cells (TEM), central TM cells (TCM), and tissue‐resident TM cells (TRM). TEM and TRM predominantly provide protective memory, displaying rapid effector functions during secondary responses. Conversely, TCM reside in peripheral lymphoid organs, do not exhibit immediate effector functions, and differentiate into effector cells upon re‐exposure to the antigen. The mechanisms underlying the enhanced secondary immune responses mediated by TM cells are not fully elucidated but may include several factors: increased TCR affinity allowing activation by lower antigen concentrations, reduced dependence on costimulatory signals, heightened cytokine production, and increased sensitivity to cytokine effects.^71^, ^72^

Regarding B cell‐mediated humoral immunity, antigen‐stimulated B cells that enter GCs ultimately differentiate into PCs and long‐lived MBCs. Upon re‐encounter with the same antigen, MBCs can mount a rapid, robust, and sustained specific antibody response without requiring T helper cell assistance. MBCs exhibit several features, including increased BCR affinity, upregulated MHC class I and costimulatory molecules enhancing antigen sensitivity, significantly higher clonal frequency and proliferation rate, and antibody levels that exceed those of the primary response by more than tenfold and last longer. Furthermore, having undergone class switching, MBCs predominantly produce high‐affinity IgG antibodies during subsequent responses.^73^, ^74^

In conclusion, the adaptive immune memory, which is mediated by T and B cells, is a complex and precisely regulated process that ensures a fast and potent immune response upon re‐exposure to antigens. This plays a critical role in long‐term immunity and host defense.

## 4 CROSSTALK BETWEEN INNATE AND ADAPTIVE IMMUNITY

In the previous sections, we briefly introduced the basic concepts of innate and adaptive immunity, including their components and primary functions. In this section, we will focus on the intricate interactions between innate and adaptive immunity. First, we will discuss the critical role of innate immunity in the activation and effector functions of adaptive immunity. Next, we will examine how adaptive immunity communicates with innate immunity through cytokine signaling. Finally, we will explore how the regulation of adaptive immunity by innate immunity contributes to the maintenance of immune homeostasis.

### 4.1 The participation of innate immunity in adaptive immunity

Innate immunity plays a key role in the initiation of adaptive immune responses. APCs are central to bridging innate and adaptive immunity. APCs use PRRs on their surface to recognize and phagocytose pathogens, processing them into pMHC complexes. These complexes are then presented to T cells, providing the initial signal required for T cell activation. Activated innate immune cells then upregulate costimulatory molecules, providing the second signal required for T cell activation. In addition, these activated cells secrete a variety of cytokines and chemokines that promote T cell activation, proliferation, and migration.^14^ In particular, DCs, as professional APCs, have been shown to induce diverse T cell effector responses. Extensive data have been generated on the functional heterogeneity of DCs, including DC subset‐specific expression of PRRs and signaling molecules that contribute to T cell differentiation into effector cells,^18^, ^75^ as well as the transcriptional master regulators of different DC lineages.^76^, ^77^

Innate immunity also contributes to the effector phase of adaptive immune responses. In humoral immunity, macrophages and NK cells can mediate pathogen clearance through opsonization and ADCC in the presence of specific antibodies.^78^ Furthermore, IgE‐sensitized mast cells and basophils mediate type I hypersensitivity reactions through degranulation.^79^ In cellular immunity, Th1 cells induce a delayed‐type hypersensitivity response, leading to extensive macrophage infiltration at the site. These activated macrophages then release cytokines, proteases, and collagenases to eliminate target antigens.^80^ Th17 cells, a crucial component of adaptive immunity, primarily exert their effects by secreting IL‐17, recruiting neutrophils, and mediating inflammation and tissue damage.^81^

### 4.2 Cytokine signaling

The interaction between intrinsic and adaptive immunity is mediated by complex cytokine signaling pathways that coordinate the immune response to pathogens and maintain immune homeostasis. On the one hand, intrinsic immune cells recognize pathogens through PRRs, initiating the production of cytokines such as IL‐1, TNF‐α, IFNs. These cytokines act as alert signals that trigger inflammation and activate adaptive immune cells. For instance, IL‐1 and TNF‐α facilitate the maturation and activation of DCs, which are pivotal for antigen presentation to T cells. Additionally, IFNs play a pivotal role in enhancing antigen presentation and promoting T cell differentiation to effector and memory cells. The defense of CD8+ T cells and Th1 cells against intracellular pathogens necessitates IL‐12, which is preferentially induced by Batf3‐dependent CD103+ DCs.^82^, ^83^ Moreover, the characterization of the initiating cytokines leading to the differentiation of the various Th lineages has been extensively investigated.^84^, ^85^ Conversely, adaptive immune cells, particularly T cells, regulate the intrinsic immune response by secreting cytokines. Th1 cells are responsible for the production of IFN‐γ, which serves to enhance the microbicidal activity of macrophages and strengthen their ability to clear intracellular pathogens. Additionally, Th2 cells secrete cytokines, including IL‐4 and IL‐13, which promote alternative macrophage activation and contribute to tissue repair and antiparasitic responses. This two‐way cytokine‐mediated communication between intrinsic and adaptive immune cells ensures the integration and optimization of immune responses against specific threats encountered by the host.^14^ Moreover, recent studies have shown that TM cells can coordinate a wide range of alterations in the innate immune response by rapidly secreting IFN‐γ.^86^

TLRs are involved in the regulation of innate and adaptive immunity, which control the activation of APCs and key cytokines.^87^ However, recent studies have shown that TLR signaling can also directly regulate adaptive immunity. This is done by modulating the development and function of T cells and B cells.^88^, ^89^ T cells express a unique combination of TLRs, and the expression of these TLRs is regulated by TCR‐dependent activation. Moreover, TLRs can act as costimulatory receptors on T cells, connecting to support TCR‐mediated signaling and costimulating cytokine production, proliferation and survival.^90^ B cells also express a nearly complete set of TLRs with associated signaling mechanisms.^91^ Similar to T cells, B cells can coordinate innate and adaptive immune functions by integrating signals via TLRs with the B cell antigen receptor or coreceptor CD40.^92^

### 4.3 Maintenance of immune homeostasis

Innate immunity regulates adaptive immune responses in various ways to maintain immune homeostasis. First, innate immunity can influence the type of adaptive immune response through cytokines. Innate immune cells recognize different types of pathogens, initiating distinct adaptive immune responses to eliminate them.^93^ For instance, activated macrophages secrete IL‐12, while activated NK cells produce IFN‐γ, promoting Th1 cell differentiation.^94^ Conversely, certain parasitic infections stimulate macrophages to secrete IL‐10, and mast cells and basophils to release IL‐4, driving Th2 cell differentiation.^95^ These processes influence the balance between Th1 and Th2 responses. Additionally, innate immunity regulates the balance between humoral and cellular immunity. NK cells enhance T cell function by releasing cytokines such as IL‐2, IFN‐γ, TNF‐α, and granulocyte‐macrophage colony‐stimulating factor (GM‐CSF), while significantly inhibiting B cell differentiation and antibody production, even killing LPS‐activated B cells to suppress the humoral response.^96^ Moreover, innate immunity affects the strength of adaptive immune responses. Mechanistically, innate immune cells express effector molecules that enhance antigen capture and presentation or lower activation thresholds. For example, innate immune cells secrete immunostimulatory factors like IL‐1, IL‐12, IL‐4, and TNF‐α to promote adaptive immune responses, while also releasing immunosuppressive factors such as TGF‐β and reactive oxygen species (ROS) to inhibit immune reactions.^97^ A recent study has identified a positive feedback loop between IL‐12 and IFN‐γ, whereby IL‐12 initiates an intrinsic feedforward loop in B cells, thereby amplifying IFN‐γ production. In conjunction with IL‐12, IFN‐γ promotes B cell proliferation and PC differentiation. In this context, IL‐12 can originate from B cells, DCs, or macrophages.^98^

Second, a recent review have indicated that the complement system can function intracellularly to guide T cell fate, adding complexity to the role of complement in regulating adaptive immunity.^99^

Third, a study proposed that MSs detect MADSs, offering a new mechanism linking innate and adaptive immune responses. They highlighted that ROS production can induce activated immune cells and adaptive immune cells, thereby regulating their activity and the interplay between innate and adaptive immunity.^100^

Finally, mast cells play a crucial role in promoting phagocytosis, antigen presentation, and pathogen clearance during the early stages of infection. In the later stages of infection, mast cells provide essential support for T and B cell activation, fine‐tuning tolerance or immunosuppression as needed.^101^

## 5 DYSREGULATION OF INNATE AND ADAPTIVE IMMUNITY

Under normal conditions, the immune system maintains the stable work of the organism. However, in the face of external pathogen attacks or after its own dysfunction, the immune system can directly or indirectly cause harm to the organism. Although the immune system is involved in almost all diseases, the following section focuses on the functions and dysfunctions of innate and adaptive immunity in three categories: infections (including viral, bacterial, and fungal), autoimmune diseases and cancer.

### 5.1 Infectious diseases

#### 5.1.1 Viral infections

During viral infection, the innate immune system serves as the first line of defense and requires an extended set of roles: pathogen perception, signal transduction, transcription, translation, protein folding, and translocation to the site of action.^102^ Pathogens are primarily perceived as targeting the genetic material DNA or RNA of viruses and are responsible for PRR, including RLRs, TLRs, AIM2, NLR (RNA sensors), IFN‐γ inducible protein 16, cGAS, and dead‐box helicase 41 (DNA sensors).^103^ These genes are activated by viral genetic material, triggering downstream pathways such as the MAVS–TBK1–IRF3 signaling,^104^ inhibiting viral replication and transmission, and inducing the activation of adaptive immune responses such as T‐expression of costimulatory/coregulatory molecules and B‐cell activation.^105^ In recent years, new ideas have emerged, suggesting that innate immune cell functionality can be influenced by previous exposures, beyond the traditional activation by direct pathogen contact. This modulation is mediated by metabolites, IFN, and cytokines triggered by these metabolites, leading to a state known as intrinsic innate antiviral immunity. Single‐cell sequencing has demonstrated that influenza vaccination in humans sets up a durable epigenetic program in monocytes, which confers resistance to subsequent in vitro viral infections.^106^ This epigenetic modification that occurs on innate immune cells is defined as trained immunity, which is heritable and can influence the strength of host immunity to pathogenic microbial infections.^107^

The role of NK cells in antiviral resistance is another research hotspot of the innate immune system in recent years. The mechanisms behind the enhanced NK cell‐mediated pathogen recognition response after viral infection may include infection‐induced upregulation of self‐encoding molecules and/or concomitant regulation of cellular stress responses and cytokines.^108^ In addition to this, NK cells can eliminate virus‐infected cells through CD16‐mediated ADCC^109^ (Table 3).

**TABLE 3:** The main antiviral effect of natural killer cells.

Viral	NK subpopulation	Mechanism	References
Flavivirus	CD56bright and CD56dim NK cells	Through type I and type III interferons	^110^
Dengue virus	NK cells	IL‐18 drives NK cell proliferative response; NK cells produce IFNγ	^111^
Influenza A viruses	CD56bright and CD56dim NK cells	Being activated	^112^
Influenza A viruses	CD16‐ CD49a+ CXCR3+ NK cells	CD49a and CXCR3 promoting homing to and tissue retention in the lungs	^112^
Seasonal influenza	NK cells	ADCC effects toward infected target cells	^113^
HCV/HBV	NK cells	Targeting activated CD4+ T cells to maintain chronic infection, which in turn leads to CD8+ T cell exhaustion	^114^
HIV‐1	NK cells	Acts as a natural ligand for CCR5 and hinders HIV infectivity in target cells	^115^
HIV‐1	KIR3DS1+ NK cells	Bw4‐80Id‐dependent inhibition of viral replication	^116^

#### 5.1.2 Bacterial infections

**FIGURE 2:**
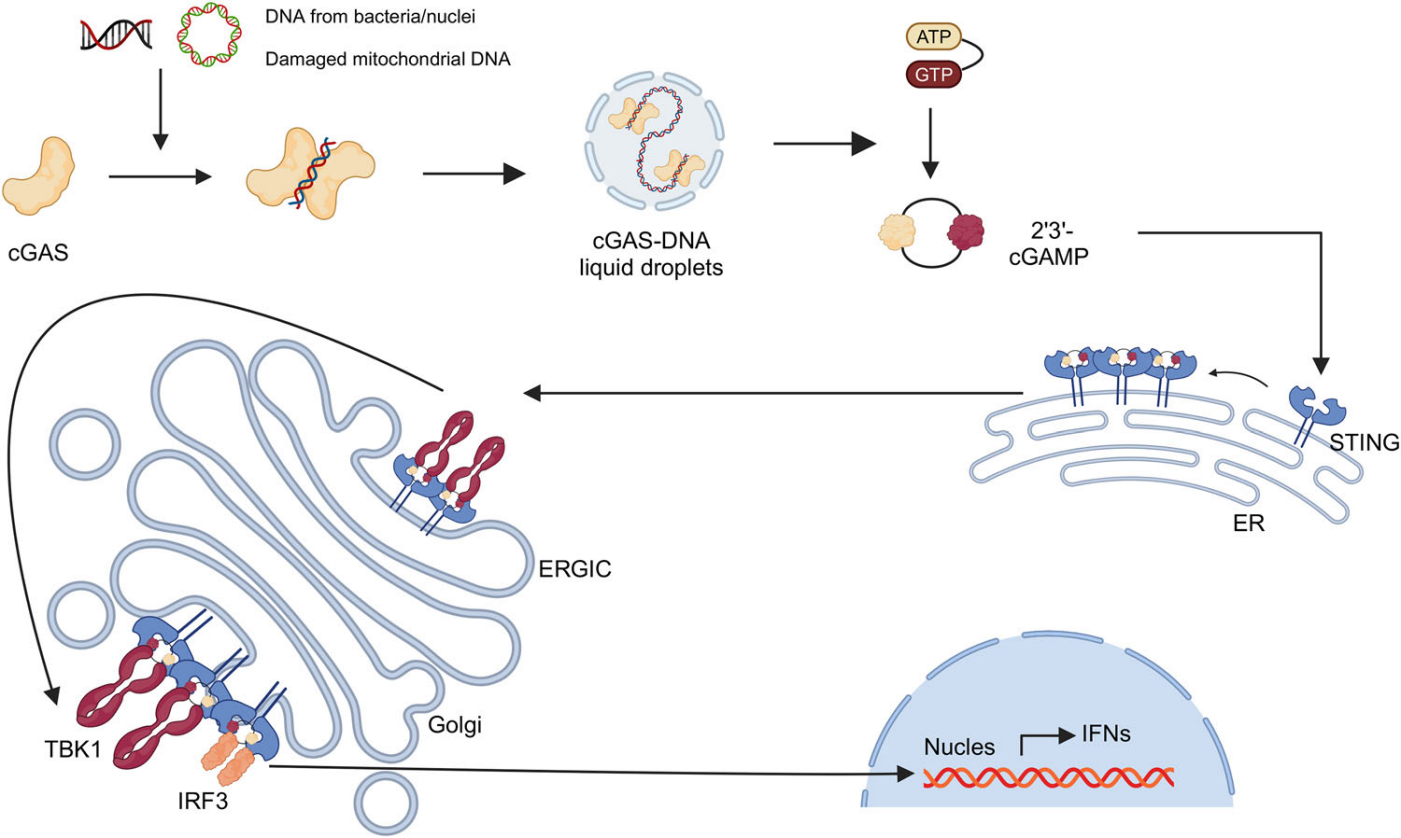
cGAS–STING pathway. The figure shows the whole process from the recognition of the infection by cGAS to the production of the effect. First, cGAS senses DNA and forms cGAS–DNA droplets. ATP and GTP in this complex are catalyzed to produce 2′3′‐cGAMP.It binds STING and initiates the transport of STING from the ER to the Golgi. During translocation, STING recruits TBK1 and IRF3.Phosphorylation of STING by TBK1 leads to phosphorylation of IRF3 and translocation to the nucleus. This is followed by transcription of IFN‐I and many other inflammatory cytokines. Created with BioRender.com.

Similar to defense mechanisms in response to viruses, the innate immune system recognizes the components of bacteria through a limited number of germline‐encoded PRR, which subsequently initiate downstream signaling leading to cytokine secretion of inflammatory factors, type I IFNs, chemokines, and AMPs. In recent years, an important component of innate immunity, cGAS and the STING, has also gradually gained attention and become a research hotspot (Figure 2).^117^ The cGAS–STING pathway is known to counteract viral infections, but its role in bacterial infections is more intricate and diverse. For example, during one of the most common infections, *Staphylococcus aureus* infection, the STING pathway can limit infection and protect lung structure and function by inhibiting necrotic apoptosis in macrophages,^118^ in addition to responding to *S. aureus* DNA.^119^ The STING pathway also impacts Gram‐negative bacterial infections. For example, it regulates *Brucella abortus* replication through metabolic reprogramming in macrophages, increasing succinate levels to stabilize hypoxia‐inducible factor 1α, which then produces proinflammatory cytokines to limit the infection.^120^

Once innate immunity is activated, the activation of adaptive immunity follows logically. Here, we will not go into details about the classic ways in which innate immunity activates acquired immunity (see above), but focus on summarizing the latest research content and directions in acquired immune responses.

In recent years, T cell research has intensely focused on bacterial T cell superantigens (SAgs), a class of microbial exotoxins that activate a substantial number of T cells and are predominantly produced by *S. aureus* and *Streptococcus pyogenes*. SAgs activate T cells through the direct binding and cross‐linking of the lateral regions of MHC class II molecules on APCs with TCRs on T cells. This mechanism differs from the typical TCR–pMHC class II activation by triggering T cells independently of TCR antigen specificity.^121^ Both CD4+ and CD8+ T cells can be activated by SAgs via MHC‐II binding,^122^ but different activated T cell subsets can influence infection differently depending on the bacterial species. For instance, in a model of nasal *S. pyogenes* colonization sensitive to SAg, the removal of CD8+ T cells provided infection protection,^123^ whereas in the SAg‐sensitive model of *S. aureus* bacteremia, removal of CD4+ T cells was protective. While SAgs typically induce a proinflammatory response, low SAg concentrations can also prompt immunosuppressive T cells and promote Treg cell responses that produce IL‐10.^124^ Treg cells appear to be stimulated by lower concentrations of SAg than proinflammatory T cells. Notably, SAg‐stimulated Treg cells also secrete IFN‐γ and IL‐17A, meaning they do not solely provide protective effects.^125^

Besides SAgs, another significant area of research focuses on the formation of MBCs. Since nearly all vaccines depend on inducing B cell memory, developing effective vaccines requires a deep understanding of the cellular and molecular mechanisms that control MBC production, function, and reactivation.^126^ MBCs are characterized by class switching of their high‐affinity surface BCRs, a process initially thought to occur exclusively within GCs.^127^ However, recent studies have revealed the existence of GC independent^128^ and unconverted MBCs,^129^ indicating that B cell differentiation into MBCs involves multiple factors. At the same time, although T cells play an important role, they are not necessary therefore. As found in the antibody response to Streptococcus pneumoniae infection, memory B1b cells persist in the peritoneal cavity, and their development is not dependent on expected interactions with T cells.^130^

#### 5.1.3 Fungal infections

Unlike viral and bacterial infections, fungal infections have not received sufficient attention in the past. However, in recent years, due to the increasing number of susceptible populations, such as immunocompromised patients undergoing chemotherapy and organ transplantation, patients treated with broad‐spectrum antibiotics or invasive medical procedures, and the emergence of drug resistance, the World Health Organization (WHO) released its first list of fungal priority pathogens at the end of 2022,^131^ highlighting the imminent need for research into fungal infections and associated treatments.

The fungal cell wall is composed of conserved β‐1,3‐glucan, β‐1,6‐glucan, and chitin, surrounded by O‐ and N‐linked mannoproteins. These components form PAMPs recognized by various PRRs such as TLR, CLR, NLR, and RIG‐I.^132^ When fungal PAMPs are detected by PRRs on phagocytes, phagocytosis is rapidly triggered, serving as the primary defense against fungal invasion. However, fungi can evade this process to survive within the host. For instance, they can switch from yeast to mycelial growth, reducing their size to avoid phagocytosis. Additionally, each fungal species has unique antiphagocytic strategies. *Clostridium perfringens* and *Candida smoothii*, for example, prevent the acidification of phagolysosomes, thereby inactivating antimicrobial and lysosomal enzymes.^133^ *Candida albicans* maintains redox homeostasis by activating the glutathione system, allowing it to evade oxidative killing within phagolysosomes.^134^ Alternatively, fungi can rupture phagocytes through invasive mycelial growth to escape.^135^

Beyond phagocytosis, the innate immune system combats fungal infections using both oxidative and nonoxidative methods. Upon fungal invasion, innate immune cells activate the NADPH oxidase complex on the cell membrane, generating high levels of ROS to kill the fungus.^136^ Fungi counteract this immune response by evolving various antioxidant mechanisms, such as using catalase to scavenge hydrogen peroxide and melanin, mannitol, and superoxide dismutase to neutralize superoxide.^137^ Additionally, AMPs like LL‐37, histatin (Hst), and defensins directly inhibit fungal growth. LL‐37, for example, disrupts membrane integrity, causing nucleotide, ATP, and protein leakage in *C. albicans*.^138^ Hst5 is taken up by *C. albicans*, where it induces ROS production and triggers a noncleavage efflux of ATP from mitochondria, leading to cell death.^139^ Defensins deplete intracellular ATP levels in fungal cells, ultimately causing their demise.^140^

After the innate immune system is activated, the acquired immune system begins to play its role. Its basic functions are similar to the model described earlier, such as DC processing and presentation of fungal antigens on MHC class I or II molecules to provide costimulatory signals, as well as secretion of specific cytokines and chemokines to regulate lymphocyte functions necessary for the control of fungal infections.^141^ However, the unique role of B cells and T cells in fungal infections has not been a breakthrough discovery. Interestingly, the role of antibodies in the course of fungal infections appears to be controversial. While secreted IgA from human breast milk has been shown to prevent *C. albicans* from binding to human oral epithelial cells,^142^ but IgA levels do not change significantly after recurrent vulvovaginal candidiasis.^143^

### 5.2 Autoimmune diseases

By definition, an autoimmune disease is a breakdown in immune self‐tolerance resulting in the adaptive immune system mistakenly attacking healthy cells, tissues and organs.^144^ Dysregulation of immune tolerance due to abnormally functioning T and B cells is central in the pathogenesis of this group of diseases. In the process of erroneous attack by T and B cells, innate immune cells also play a supporting role, further aggravating tissue and organ damage.

#### 5.2.1 Dysregulation of immune tolerance

As previously explained, tolerance is a state of immune unresponsiveness that primarily involves B and T cells and is divided into central and peripheral components.^145^, ^146^ In both regions, the interaction of the cells with antigens generates a process of negative selection, which determines the fate of the cells. From the current findings, it is clear that disturbances in immune tolerance are the result of a combination of multiple factors, including genetic mutations, pathogenic microbial infections, smoking, drugs, and pregnancy.^147^

In response to these pathogenic factors, B cells secrete antibodies that bind to the body's own tissues and destroy them. There are three main ways in which they can cause disease. First, autoantibodies can alter cell function by binding directly to cell surface receptors through their Fab or Fc. For example, in Graves' disease, antibodies targeting the thyrotropin receptor act as agonists, whereas in myasthenia gravis, antibodies against the acetylcholine receptor serve as antagonists. Second, autoantibodies can disrupt physiological processes through the complement system or antibody‐dependent cytotoxic elimination of cell populations. Last, a key mechanism involves the formation of immune complexes with antigens.^148^ These complexes can deposit in tissues, activating complement and driving inflammation by recruiting neutrophils and other immune cells to the affected areas.

Although autoantibodies are crucial in autoimmune diseases, the role of T cells is equally important. In the normal immune system, CD4+ T cells help stimulate B cells to produce autoantibodies, while cytotoxic CD8+ T cells can directly damage or kill the body's own cells. In disease states, however, Treg cells transition from an immunosuppressive state to an effector T cell state. This shift has been observed in conditions such as multiple sclerosis, inflammatory bowel disease, systemic lupus erythematosus (SLE), and rheumatoid arthritis (RA), although the mechanisms behind this change are not yet clear.^149^ Additionally, T cells can produce factors, such as circulating permeability factor, which can contribute to diseases like focal segmental glomerulosclerosis. However, the vast diversity in T cell antigen recognition and the molecules encoded in the MHC region makes determining T cell autoreactivity a significant technical challenge. Consequently, T cells in autoimmune diseases have been poorly studied. Notably, changes in the physiological state of tissues or organs, based on T cell autoreactivity, can influence autoimmune injury. For example, destruction of a subset of pancreatic β‐cells in IDDM increases cellular stress in the remaining β‐cells, which increases their susceptibility to auto‐reactive T cell injury, resulting in a positive feedback acceleration.^150^

#### 5.2.2 Role of innate immune activation

Although adaptive immunity plays a direct role in autoimmunity, the role of innate immunity in disease should not be underestimated because of its ability to interact with and activate adaptive immunity. Tolerant DC subpopulations have been found to attenuate the progression of autoimmune disease in mice by promoting Treg cell expansion and inducing autoreactive lymphocyte unresponsiveness.^151^ Another important innate immune cell is the macrophage, which suppresses the activity of autoreactive B cells by releasing CD40L and IL‐6. However, this process has not been observed in autoimmune conditions.^152^ Granular immune cells, such as neutrophils, contribute to the development of autoimmune disorders.^153^ These cells are highly prevalent in the bloodstream, exhibit phagocytic activity, generate ROS, and participate in the creation of neutrophil extracellular traps (NETs). NETs have been found in pathological conditions of autoimmune diseases such as RA and MS.^154^ In addition, NETs can act as antigens that bind to autoantibodies to produce immune complexes that activate PC‐like DCs and induce IFN‐α secretion.^155^

In addition to the traditional innate immune cells, recent findings suggest that trained immunity, a memory‐like feature in innate immune cells, is associated with autoimmune diseases. In SLE, injecting β‐glucan into mice worsened the disease by promoting IL‐1β secretion and enhancing glycolysis in innate immune cells.^156^ Elevated levels of proinflammatory cytokines in patients,^157^ such as IL‐6, TNF‐α, and IL‐1β, were comparable to those observed in trained immunity.^158^ However, in RA, β‐glucan is able to increase the secretion of TNF‐α from innate immune cells and thus improve clinical scores in mice. Similarly, in T1D, β‐glucan has also been suggested as a novel therapeutic strategy because of its ability to protect the organism by activating the innate immune response in nonobese diabetic (NOD) mice,^159^ thereby protecting the organism.^160^

### 5.3 Cancer

Cancer, as a systemic disease, induces many functional and compositional changes throughout the immune system, the first of which occurs as a result of dysregulation of the immunosurveillance role. As the disease progresses, the overall immune landscape beyond the eventual tumor is significantly altered. The classification of cancers into solid and hematologic cancer can help us to better understand the role that the immune system plays in the pathogenesis of cancer.

#### 5.3.1 Immunity of solid tumor: the cancer‐immunity cycle

**FIGURE 3:**
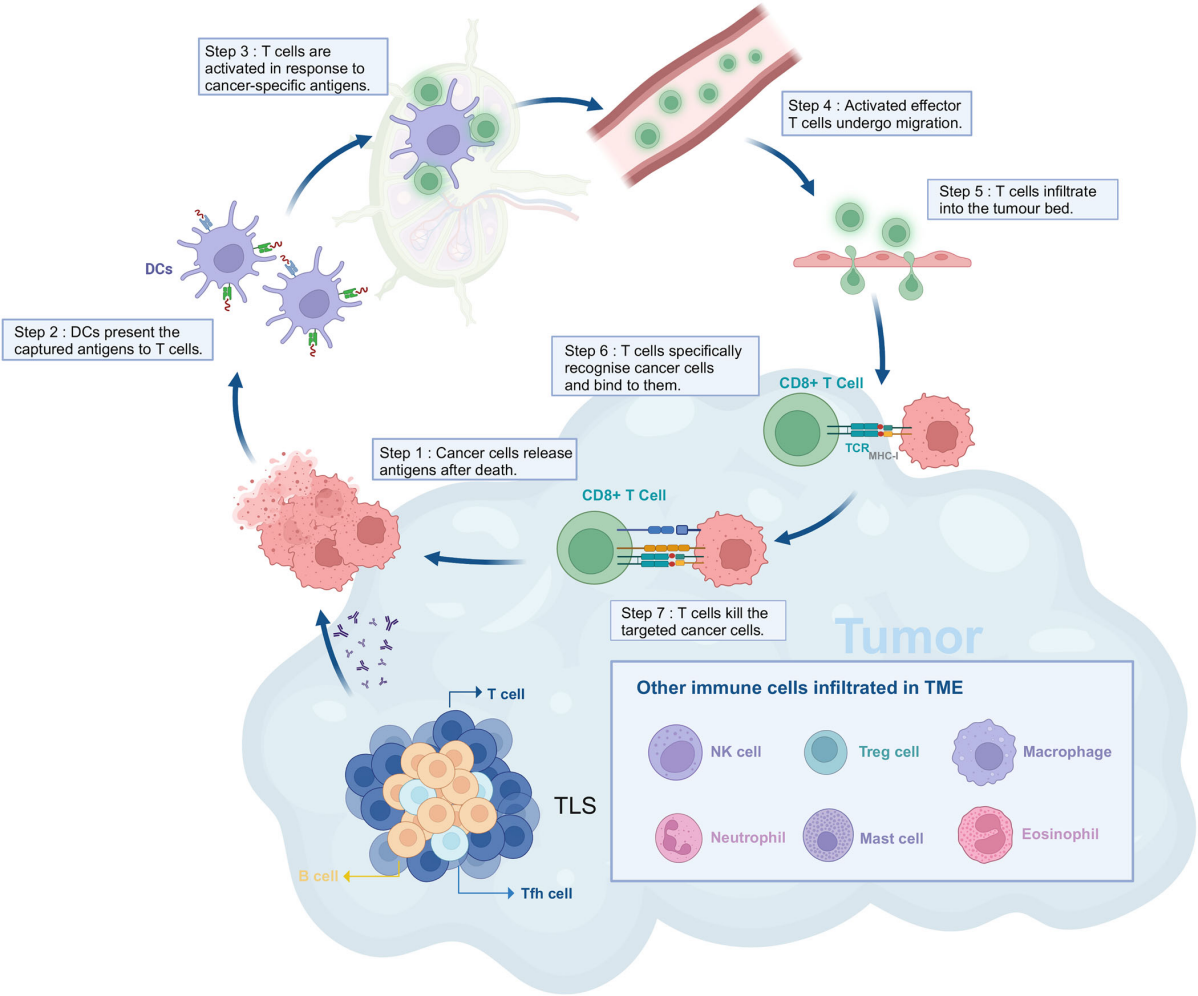
The cancer‐immunity cycle and TME. The figure shows the components of cancer immunity. The upper part shows the core of cancer immunity, a cyclical process of accumulation of immunostimulatory factors and amplification of the killing effect. This cycle can be simplified into seven steps, starting with the release of antigens from the death of cancer cells and ending with the killing of cancer cells by T cells. The other components of the TME are shown below. The TLS has an inner zone of CD20+ B cells surrounded by T cells, similar to the structure of lymphoid follicles in secondary lymphoid organs. Created with BioRender.com.

Although the role of the immune system, and in particular immune surveillance, in cancer has been studied for decades, clinical observations and immune‐therapy in recent years have reshaped the way researchers understand cancer immunity (CI). To better integrate clinical care, in 2013 Daniel S. Chen and Ira Mellman introduced the concept of the CI cycle: immune cells neither respond nor function on their own, but rather exist in the context of a series of steps.^161^ These steps interact with each other, and any single step has the potential to limit the rate at which optimal immunity is produced. In detail, the cancer immune cycle is divided into seven steps. First, antigens generated by tumor cells are released and taken up by DCs for processing. Next, the antigen is presented to T cells. These molecules are perceived as foreign or inadequately tolerated, and can thus trigger and activate effector T cell‐specific immune responses against cancer cells. Finally, the activated effector T cells move and infiltrate toward the tumor site, specifically identifying and binding to the cancer cells, and subsequently destroying them. Dead cancer cells release additional tumor‐associated antigens, thus returning to the initial step and amplifying the immune response in subsequent cycles (Figure 3).^162^

Furthermore, the efficiency of this cycle differs markedly in different tumors and is mainly determined by the immunophenotype of the tumor. The three classical immunophenotypes—immunoinflammatory, immune‐excluded, and immune‐deserted—are defined as follows: tumors with abundant immune infiltration, tumors where T cell infiltration is restricted to the tumor mesenchyme rather than the parenchyma, and tumors lacking immune infiltration, respectively.^163^ Although this classification is now generally accepted and more commonly used, it has been found to be an oversimplification as studies have been carried out. The three classical immunophenotypes—immunoinflammatory, immune‐excluded, and immune‐deserted—are defined as follows: tumors with abundant immune infiltration, tumors where T cell infiltration is restricted to the tumor mesenchyme rather than the parenchyma, and tumors lacking immune infiltration, respectively. The tumor microenvironment (TME) comprises not only T cells, but also cells of the innate immune system, B cells, and nonimmune cells like cancer‐associated fibroblasts. On the one hand, these cells can work together to inhibit T cell function and physically limit their migration into the tumor nests to limit T cell immunity.^164^ On the other hand, TME can also promote the formation of peripheral lymphoid aggregates and tertiary lymphoid structures, which can more rapidly produce more specific immune cells and thus suppress tumor growth.^165^ In summary, most of these cells have dual roles in tumors, and a recently published review describes them in more detail.^166^

#### 5.3.2 Immunity of hematologic malignancy

Hematological malignancies originate in the lymphohematopoietic system and include mainly acute leukemia, chronic leukemia, lymphoma, and multiple myeloma (MM).^167^ The cancer‐immunity cycle can also be applied to hematologic malignancy. However, unlike solid tumors, immune and cancer cells are dispersed throughout the hematopoietic system and are in constant contact with each other, favoring an environment of immune surveillance. Moreover, since tumors have the same cellular origin as the immune system, these cancer cells have a stronger immunostimulatory effect.^168^

In the innate immune system, tumor‐associated macrophages (TAMs) play an important role in tumor suppression. In adult AML, there is an increased proportion of anti‐inflammatory M2‐like macrophages and a decreased proportion of proinflammatory M1‐like macrophages in the bone marrow.^169^ Cancer cells are able to evade immune surveillance by expressing signaling ligands such as the integrin‐related protein CD47. Specifically, CD47 is able to bind to receptor signal‐regulated protein α (SIRPα) on macrophages, thereby blocking macrophage phagocytosis.^170^ Similar to macrophages, circulating DCs can also be divided into two functionally distinct subsets: conventional DCs (cDCs) and unconventional DCs, the latter of which include pDCs.^171^ Studies have shown that cDCs can capture and cross‐present tumor antigens, helping to inhibit tumor growth,^172^ while high levels of pDCs are negatively correlated with overall survival following allogeneic hematopoietic stem cell transplantation.^173^ In addition to DCs and macrophages, NK cells, mast cells and neutrophils play an auxiliary role in the development of tumors.^174^

From the perspective of adaptive immunity, T cell attacks on hematological tumors have been intensively studied. During tumor pathogenesis, T cell tolerance is controlled centrally, and APCs may cause immunodeficiency when they first encounter antigens from their own blood system.^175^ Acquired T cell dysfunction arises from the interaction between myeloma cells and T cells, leading to impaired T cell immunity against the tumor, while the response to external antigens remains largely unaffected.^176^ Unlike solid tumors, clonally expanded T cells are found in the blood of myeloma patients, but these T cells are more functionally senescent than exhausted.^177^

However, similar to solid tumors, B cells cannot directly kill tumor cells, so they have received less attention than T cells. Based on current research results, the role of B cells in hematological tumors is not clear.

## 6 THERAPEUTIC IMPLICATIONS

Based on the understanding of the disease as well as the immune system, current research in therapeutic directions focuses on two main areas. One is host‐targeted therapies, which disrupt host cell processes essential for pathogen survival or replication, or modify the host's immune response to the infection, such as by boosting the activity of immune cells. The second is vaccines, which have a relatively longer history of research. Vaccines can stimulate a stronger host immune response by mimicking the pathogen and have gained widespread use. However, even with decades of research, vaccines against certain infectious diseases are still difficult to develop.

### 6.1 Immunomodulatory drugs

Immunomodulatory drugs are a type of medication that treats disease by regulating the strength of the body's own immune system response. There are two main types of treatment: immunostimulants and immunosuppressants. The former are mainly used in the treatment of infectious diseases, tumors, and primary or secondary immunodeficiencies, while the latter are mainly used to reduce the immune response to transplanted organs and in the treatment of autoimmune diseases.^178^ Compared with traditional treatments such as chemotherapy, radiotherapy, and hormone therapy, immunomodulators regulate the function of the immune system more accurately, resulting in more pronounced efficacy and fewer side effects that can harm other systems.

#### 6.1.1 Targeting innate immunity

There are two main types of immunomodulators targeting innate immunity therapy, one intervening in the classical pathways and cytokines of innate immunity and the other modifying innate immune cells.

Interferon has long been considered a powerful tool in the treatment of infections. It is secreted by leukocytes and is able to interfere with viral replication and activate immune cells such as NK cells and macrophages.^179^ Type I interferon has shown promising antiviral effects in patients infected with HCV, influenza, and SARS‐CoV‐2. Notably, the primary time point of action was early in the course of viral infection, as demonstrated in preclinical studies of influenza, severe acute respiratory syndrome (SARS), Middle East respiratory syndrome (MERS), and COVID‐19.^180^ A retrospective multicohort study showed that early treatment with IFNα improved the chances of survival, whereas later initiation of interferon therapy reduced the chances of survival.^181^ The WHO Solidarity Trial also showed that IFNβ1α slightly increased the risk of death in patients requiring supplemental oxygen therapy.^182^ In response to these experimental results, a possible explanation relates to the cellular specificity of the interferon receptor. Receptors for type I interferons are widespread and may drive inflammation and immunopathology in later stages of the disease.^183^ Conversely, the receptors for type III interferons are mainly found in epithelial cells. Therefore, IFNλ reduces certain side effects compared with type I interferons and is recommended as the first choice for interferon therapy.^184^

In addition, inflammatory storm is one of the main causes of death from severe infections, and therefore anticytokine approaches are a current research priority.^185^ Among them, IL‐6‐related studies have made some progress, such as anti‐IL‐6 therapies have been successful in the treatment of RA and the highly inflammatory complications of CAR T cell therapy.^186^ Two classes of anti‐IL‐6 agents have been investigated, including tocilizumab and sarilumab, which target the IL‐6 receptor, and siltuximab, which targets IL‐6 itself. Similarly, after transplantation, IFN‐γ can cause injury by increasing Fas expression in hepatocytes and acting synergistically with IL‐18. Therefore, the addition of an inhibitory cytokine active ingredient to perfusion therapy may enhance the immune microenvironment and reduce the incidence of transplantation complications.^187^ Apart from that, therapies to block other cytokine signals such as GM‐CSF and IL‐1, as well as Janus kinases, which are downstream signals of cytokine receptors, have also progressed.^188^

In the tumor microenvironment, although T cells play a major killing role, innate immune cells are also infiltrated, influencing tumor development as well as therapeutic outcome. Recent studies have focused on DCs, NK cells, myeloid‐derived suppressor cells (MDSCs) and macrophages.^189^

Currently, the main functional regulators of DCs include agonists and immunosuppressive blockers. Experiments have shown that STING agonists upregulate costimulatory molecules and MHC on DCs, and improve antigen presentation.^190^ TLR3 agonists promote cDC1 maturation and produce large amounts of cytokines.^191^ Immunosuppressive signaling includes various factors such as TGF‐β, IL‐10, IDO, PGE2, and VEGF, which impair DC function, disrupt immune surveillance, and facilitate tumor progression.^192^ Evidence indicates that anti‐VEGF antibodies enhance DCs function in the spleen and lymph nodes, synergize with peptide‐pulsed DCs, and extend survival in hormonal mice.^193^ The use of anti‐IDO siRNA therapy improved the cytokine production and antigen presentation abilities of DCs.^194^

In TME, activated NK cells can destroy tumor cells by inducing apoptosis through the release of perforin and granzyme, as well as ADCC, FasL, or TRAIL.^195^ Additionally, NK cells secrete cytokines, including IFN‐γ and TNF‐α, which lead to tumor growth inhibition.^196^ Since the 1980s, NK cell pericyte transfer techniques have been employed to treat hematological malignancies.^197^ In a phase 1 clinical trial, 40% of MM patients achieved clinical remission after receiving two NK cell infusions.^198^ Because of its greater safety profile relative to other over‐the‐counter cell transfer therapies,^199^ investigators have positioned NK over‐the‐counter cell therapy as a therapeutic equivalent to CAR‐T.^200^ The latest advancement in NK cell relay cell transfer technology is chimeric antigen receptor (CAR)‐engineered NK (CAR‐NK) cells.^201^ In a phase 1/2 clinical study of anti‐CD19 CAR‐NK for the treatment of lymphoid tumors, the remission rate was 73% with no significant side effects observed.^202^ In addition, IC inhibitors targeting NK cells can enhance NK cell activation and cytotoxicity. Currently known ICs on NK cells include NKG2A/CD94, the KIR family, LIR1, T cell Ig and immunoreceptor‐tyrosine‐based inhibitory‐motif domain (TIGIT)/CD96, B7H3, programmed death‐1 (PD‐1), CTL‐associated antigen 4 (CTLA‐4), lymphocyte activation gene 3 (LAG‐3), T cell Ig and mucin domain‐containing protein 3 (TIM‐3), CD200R, and SIRPα.^203^ Related studies are ongoing, such as the anti‐inhibitory KIR antibody IPH2101 (1‐7F9), which effectively induces NK cell‐mediated killing in a mouse model of MM.^204^

Under physiological conditions, myeloid cells differentiate into mature subpopulations, but due to interference by TME, some myeloid cells differentiate into MDSC in an immature state.^205^ MDSCs have immunosuppressive properties that can promote cancer progression by promoting immune escape and treatment resistance. Therapeutic strategies targeting MDSCs can be divided into four categories: (1) inhibiting the recruitment and expansion of MDSCs; (2) promoting the differentiation of MDSCs into mature myeloid cells; (3) inhibiting the function of MDSCs; and (4) directly eliminating MDSCs.^206^ CXCLs–CXCR1/2 blockers exhibit antitumor activity in various mouse models by blocking the migration of PMN‐MDSCs to the TME.^207^ In addition, MDSCs express S100A8/A9 and its receptor, RAGE, which promotes the recruitment of MDSCs and enhances their immunosuppressive capacity. S100A8/A9 inhibitors disrupt this signaling circuit, reducing MDSC aggregation and retarding tumor growth.^208^ All‐trans retinoic acid (ATRA) is able to regulate cell differentiation, proliferation, and apoptosis via nuclear retinoic acid receptors.^209^ On this basis, researchers have found that in patients with metastatic renal cell carcinoma (RCC), ATRA significantly reduces MDSC and increases cDC/pDC ratio in peripheral blood.^210^ The COX‐2–PGE2 axis is crucial for maintaining the immunosuppressive function of MDSC, thus celecoxib (COX‐2 inhibitor) reduces ROS and NO production in MDSC.^211^ Some chemotherapeutic agents such as carboplatin and paclitaxel, selectively eradicate regulatory immune cells, particularly MDSCs, and attenuate immunosuppression,^212^ whereas Fc‐engineered anti‐CD33 antibody (BI 836858) and anti‐CD33 antibody‐coupled drug (gemtuzumab ozogamicin) specifically eliminate MDSC.^213^

Macrophage therapies mainly include their use as transporters as well as CAR‐M therapies. Since peripheral circulating monocytes continuously replenish the TAM reservoir within the TME, monocytes can serve as carriers to deliver therapeutic agents to the tumor.^214^ Nanoparticle‐loaded monocytes have enhanced antitumor activity compared with free nanoparticles.^215^ Similar to CAR‐T cells, CAR‐M includes extracellular antigen recognition, transmembrane, and intracellular structural domains.^216^ The initial CAR‐M product, developed in 2018 and originally termed CAR phagocytic cells (CAR‐Ps), utilized lentiviral vectors to insert CARs featuring Megf10 or FcRγ as intracellular domains into mouse macrophages.^217^ Experiments have shown that anti‐HER2 CAR‐M cells not only exhibit tumor killing capacity but also induce proinflammatory TME. Specifically, CAR‐M can boost tumor‐specific T cell activity by producing proinflammatory chemokines and cytokines, converting M2‐like macrophages to M1‐like macrophages, and increasing the expression of antigen‐presenting mechanisms.^218^ Excitingly, CAR‐M offers advantages over CAR‐T cells in solid tumors, particularly in enhancing cell trafficking and infiltration within the TME.^219^ The nanocomplexes produced by combining these two strategies consist of nanocarriers designed for macrophage targeting and plasmid DNA encoding CAR–IFN‐γ. Upon entry into the body, the complex induces the development of CAR‐M1 macrophages, which participate in CAR‐mediated phagocytosis of cancer cells and orchestrate antitumor immunoregulatory responses to prevent solid tumor growth.^220^

#### 6.1.2 Enhancing adaptive immune responses

Immunomodulators targeting the adaptive immune system include therapies targeting ICs and engineered immune cells. The main targets of the former mainly include PD‐1/programmed death ligand 1 (PDL‐1), CTLA‐4, LAG‐3, Tim‐3, and TIGIT.^221^ The latter term engineered immune cells mainly refers to the modification of T cells (Table 4).

**TABLE 4:** Key United States Food and Drug Administration approved immune checkpoint therapies.

Agent	Target	Main indication	Year of first approval
Pembrolizumab	Anti‐PD‐1	1, melanoma; 2, NSCLC; 3, HNSCC; 4, cHL; 5, PMBCL; 6, UC; 7, MSI‐H/dMMR solid tumors; 8, first/second line MSI‐H/dMMR CRC; 9, GC/GEJ; 10, esophageal cancer; 11, CC; 12, HCC; 13, BTC; 14, MCC; 15, RCC; 16, EC; 17, TMB‐H solid tumor; 18, CSCC; 19, TNBC; 20, adult cHL and adult PMBCL added new 400 mg every 6 weeks dosing regimen	2014
Nivolumab	Anti‐PD‐1	1, melanoma; 2, NSCLC; 3, MPM; 4, RCC; 5, ee; 6, HNSCC; 7, UC; 8, CRC; 9, HCC; 10, esophageal carcinoma; 11, GC/GEJ, carcinoma of the gastro‐esophageal junction and esophageal adenocarcinoma	2014
Relatlimab+Nivolumab	Anti‐PD‐1+anti‐LAG3	Melanoma	2022
Atezolizumab	Anti‐PD‐L1	1, UC; 2, NSCLC; 3, SCLC; 4, HCC; 5, melanoma	2016
Durvalumab	Anti‐PD‐L1	1, NSCLC; 2, SCLC; 3, BTC (imminent approval); 4, HCC(possible approval)	2017
Dostarlimab‐gxly	Anti–PD‐1	1, EC; 2, solid tumors	2021
Ipilimumab	Anti‐CTLA‐4	1, melanoma; 2, RCC; 3, CRC; 4, HCC; 5, NSCLC; 6, MPM; 7, ESCC	2011
Avelumab	Anti‐PD‐L1	1, MCC; 2, UC; 3, RCC	2017
Cemiplimab	Anti–PD‐1	1, SCC of the skin; 2, BCC; 3, NSCLC	2018
Tremelimumab	Anti‐CTLA‐4	1, primary HCC; 2, NSCLC	2022
Retifanlimab‐dlwr	Anti–PD‐1	MCC	2023
Toripalimab	Anti–PD‐1	1, melanoma; 2, NPC; 3, UC; 4, combination chemotherapy NPC; 5, combination paclitaxel/cisplatin ESCC; 6, combination chemotherapy for advanced NSCLC; 7, combination chemotherapy for perioperative early‐stage NSCLC. 8, combination chemotherapy for RCC	2018

Abbreviations: BCC, basal cell carcinoma; BTC, biliary tract carcinoma; CC, cervical cancer; cHL, classical Hodgkin's lymphoma; CRC, colorectal cancer; CSCC, cutaneous squamous cell carcinoma; EC, endometrial cancer; ESCC, esophageal squamous cell carcinoma; GC/GEJ, gastric and gastro‐esophageal cancers; HCC, hepatocellular carcinoma; HNSCC, head and neck squamous carcinoma; MCC, merkel cell carcinoma; MPM, malignant pleural mesothelioma; MSI‐H/dMMR, microsatellite instability‐high or mismatch repair deficient; NPC, nasopharyngeal carcinoma; NSCLC, non‐small cell lung cancer, PMBCL, primary mediastinal large B‐cell lymphoma; RCC, renal cell carcinoma; SCC, squamous cell carcinoma; SCLC, small cell lung cancer; TMB‐H, tumor mutational burden‐high; TNBC, triple negative breast cancer; UC, uroepithelial carcinoma.

*Data sources*: United States Food and Drug Administration Drugs website.

PD‐1 is an inhibitory checkpoint molecule present in T cells.^222^ It is involved in inhibiting TCR signaling by recruiting protein tyrosine phosphatases 1 (SHP‐1) and 2 (SHP‐2) tyrosine phosphatases containing Src homology 2 structural domains, which dephosphorylate molecules involved in TCR signaling like CD3ζ and ZAP‐70. Preclinical studies have shown that PD‐1: PD‐L1 binding impairs antitumor T cell responses,^223^ blocking this interaction with anti‐PD‐1/PD‐L1 antibodies enhances T cell‐mediated antitumor responses.^224^ Clinical trials have demonstrated significant efficacy of anti‐PD‐1 and anti‐PD‐L1 antibodies in patients with a variety of tumor types, including melanoma, RCC and non‐small cell lung cancer (NSCLC).^225^ Although anti‐CTLA‐4 therapies, like anti‐PD‐1/PD‐L1 therapies, inhibit signals that suppress T cell function to exert their antitumor effects, their mechanisms of action differ. CTLA‐4 primarily modulates APC‐induced T cell responses by blocking CD28‐B7 interactions. Anti‐CTLA‐4 mainly influences CD4+ T cell clonal expansion and trafficking,^226^ while anti‐PD‐1/PD‐L1 primarily targets exhausted CD8+ T cells.^227^ Clinical trials of the human monoclonal anti‐CTLA‐4 antibody ipilimumab have revealed that patients with advanced melanoma receiving this treatment exhibit durable clinical responses and long‐term survival benefits of up to 10 years.^228^ LAG‐3 is highly expressed on activated T cells and enhances Treg function and inhibits the effector function of T cells. Therefore, administration of anti‐LAG‐3 antibody may improve T cell‐mediated antitumor immunity in preclinical models.^229^

CAR T cell therapy is a type of adoptive cell therapy (ACT) that combines the potency of T cells with the specificity of antibodies to target and kill abnormal cells in a non‐MHC‐restricted manner. Single‐chain variable fragments on the cell surface confer specificity, while intracellular signaling domains activate T cell‐mediated cytotoxicity.^230^ While the first‐generation CARs consisted of a combination of CD4 extracellular domains and CD3ζ signaling domains, the second‐generation CARs added costimulatory structural domains (e.g., CD28 or 4‐1BB) for enhanced potency. Compared with conventional therapies, CAR T cells are more precise and efficient, while also being able to expand or remain on patrol in vivo depending on the number of antigens.^231^

A total of six CAR products have been approved for marketing by the United States Food and Drug Administration (US FDA), four for B‐cell lymphoma, two for B cell acute lymphoblastic leukemia (B‐ALL), and two for MM.^232^ A total of 10 studies of CD19‐targeted CAR T cell therapies for the treatment of B‐cell lymphomas provided follow‐up data for more than two years. These data show ORR of 44−91% and complete response (CR) of 28−68%.^233^ ^(p001)^ The two commercially available CAR T cell therapies for adult B‐ALL patients, tisagenlecleucel and brexucabtagene autoleucel, both showed a CR) rate of 69% in initial studies. Tisagenlecleucel had a median event‐free survival of 5.6 months and a median follow‐up of 13 months, while brexucabtagene autoleucel demonstrated a median relapse‐free survival of 7 months and a median follow‐up of 22 months.^234^ Notably, long‐term follow‐up of the currently marketed BCMA‐targeted CAR T cell therapy, ciltacabtagene autoleucel, revealed that an increasing number of patients experienced disease progression over extended monitoring periods.^235^ This suggests that patients remain at risk of disease progression over time.

### 6.2 Vaccines

Vaccines have played a vital role in preventing disease since their invention in the 18th century. Traditional vaccines include inactivated, live attenuated, and protein subunit vaccines, while new approaches in vaccinology include nonviral vaccination techniques and viral vector platforms.^236^ As the immune system has been studied in depth, today's vaccines are also more efficient and long‐lasting. This has been achieved through the development of new vaccine components, the discovery of new vaccine targets and the production of innovative adjuvants.

#### 6.2.1 Importance of priming both innate and adaptive immunity

In conventional wisdom, developers have placed more emphasis on the ability of a vaccine to activate adaptive immunity, particularly the strength of the production of specific antibodies or CD8+ cells. However, with the development of new vaccine technologies and the prevalence of COVID‐19, researchers are finding that activating innate immunity is just as important.

Adenovirus vaccines work by altering the adenovirus genome to produce nonreplicating viral particles capable of carrying the desired transgene, thereby inducing a protective immune response.^237^ After vaccination with multiple adenoviral vectors in preclinical models, type I IFN responses appear rapidly and their enhanced response levels can lead to reduced transgene expression, reduced antigen‐specific antibody responses, and reduced CD8+ T cell responses.^238^ Correspondingly, type I IFN and STING activation reduce transgene expression in mice vaccinated with adenovirus vaccines, and the amount and duration of transgene expression is the best predictor of CD8+ T cell response.^239^ Another study indicated that activating certain innate responses can positively influence the development of CD8+ T cell responses. Although CD8 T+ cell responses were not reduced in mice lacking other single TLRs, IL‐1R, or IL‐18R, a significantly reduced acquired immune response was found in MyD88 knockout mice.^240^ This suggests that no single sensing mechanism alone is responsible for activating innate immune signaling, but rather the integration of multiple mechanisms. Minimal innate sensing is necessary to generate adaptive responses, but excessive proinflammatory signaling inhibits these responses.^241^

Another example that emphasizes the activation of both innate and adaptive immunity is mRNA vaccines. It uses a lipid nanoparticle delivery platform to deliver nucleotide‐modified mRNAs encoding specific antigenic proteins into host cellular cells, which utilize the host's translation system to express the antigenic proteins.^242^ Research demonstrates that mRNA vaccines promote strong type 1 IFN signaling and DC maturation, enhancing the activation of effector T cells and B cells.^243^ Interestingly, however, blocking IFN‐α/β signaling (early type 1 IFN response) maximizes RNA replicon amplification and protein expression, leading to robust CD8+ T cell response.^244^ This suggests that the effectiveness of type 1 IFN signaling in T cell immunity triggered by antigens is time‐sensitive.^245^ Enhanced proliferation and differentiation of CD8+ T cells occur when their receptor activation precedes IFN‐α receptor signaling. At the same time, key genes for T cell memory are consistently expressed.^246^ In contrast, when IFN‐α/β begin to function before TCR signaling activation in CD8+ T cells, T cell is suppressed.^247^ Therefore, how to regulate the response sequence of innate and adaptive immunity is also a future direction for vaccine development.

#### 6.2.2 Novel vaccine strategies exploiting innate‐immune interactions

The innate immune system is gaining attention in the field of vaccine development due to its complexity and importance. One important area of research is the development of new and improved vaccines for the elderly and immunocompromised using the current understanding of innate immunity.^248^ These populations are more susceptible to infectious diseases, cancers, and poorer response to vaccination due to aging or impaired immune function. Systematic studies indicate that the innate immune response is weakened in older adults. Specifically, in the elderly, monocytes and DCs have a diminished capacity to respond to TLR ligands, and various innate immune cells fail to produce cytokines and express costimulatory factors necessary for T and B cell activation.^249^

Another area of R&D related to innate immunity is vaccine adjuvants. A large proportion of currently marketed vaccines require three or more doses to obtain antigen‐specific antibody titers sufficient to provide protection.^250^ In order to accelerate the vaccine response, a viable approach for the rapid initiation of innate and adaptive immune responses is coordination. Theoretically, adjuvants act as PAMPs that activate APCs to secrete local chemokines, accelerating the recruitment process of monocytes and neutrophils, which in turn leads to the activation of B and T cells at an early stage. A study showed that the adjuvant MF59 activated tissue‐resident macrophages. An increase in chemokines such as CCL‐2, CCL‐3, CCL‐4, and CXCL‐8 was observed at the injection site, accelerating the interaction of antigen with APCs.^251^ In another study, the TLR5 ligand adjuvant Salmonella typhimurium flagellin fljb (stf2) was shown to form a recombinant protein vaccine with the model antigen ovalbumin (OVA). Serum levels of OVA‐specific IgG were significantly increased in mice 7 days after vaccination compared with controls.^252^ Besides, the targets of adjuvants include STING pathway and MyD88‐dependent pathway, which are also currently under investigation.^250^

## 7 FUTURE DIRECTIONS

Although the immune system has long been a hot topic of biological research, there are still a large number of unanswered questions that need to be solved due to the complexity of the system and its close relationship with a wide range of diseases. It is believed that with the help of the latest technologies, such as spatial transcriptomes and artificial intelligence, breakthroughs in the field of immunology will be achieved in the future. This section summarizes future research directions in immunology at both the physiological and pathological levels.

### 7.1 Advances in understanding immune regulation

As research into innate and adaptive immunity continues to deepen, the connections between the two systems are becoming increasingly intricate, with their boundaries progressively blurring. A recent forward‐looking review explores unconventional immunological perspectives, offering new insights into the relationship between innate and adaptive immunity.^253^ Immune activation remains a central issue in immunology research. Different types of immune responses, such as antigen‐specific versus innate immune responses, primary versus memory responses, and Th1/Th2/Th17 responses, require distinct activation signals and exhibit varying levels of complexity. Our current understanding of these immune response rules remains incomplete. The authors propose that the immune system requires information from multiple channels to mount the most appropriate response. The amount of information required for an immune response is determined by the cost ratio of false positive and false negative errors. This perspective challenges the traditional distinctions between innate and adaptive immune recognition. To fully understand immune activation, it is essential to consider a variety of signals encoded in factors ranging from microbial patterns to antigen structures and dynamics. The immune system should be conceptualized as a system with collective intelligence, whereby communication and cooperation between cells enhance overall adaptability and efficiency. Shifting from traditional single‐cell cross‐sectional studies to collective cell behavior research will further our understanding of immune principles.

The advent of new technologies has opened up avenues for high‐throughput analysis of immune responses that were previously unfeasible. To illustrate, single‐cell RNA sequencing (scRNA‐seq) is capable of measuring thousands of transcripts and integrating them with protein expression data and spatial information derived from technologies such as spatial transcriptomics. The integrated application of these multiomics techniques necessitates a proficiency in computational and systems biology to facilitate the elucidation of biological insights and the identification of disease‐relevant targets. The convergence of experimental, computational, and technological fields, coupled with the emergence of data science, will propel the next phase of adaptive immunity research and stimulate novel advancements.

There remain numerous unresolved questions in the field of immune regulation that warrant further exploration. First, the specific roles of various components within the immune system require detailed investigation. For instance, the functions of recently discovered ILCs need to be elucidated, with a focus on their phenotypic spectra across different tissues and disease states. Understanding these aspects will be crucial for leveraging this knowledge in disease treatment and enhancing human health. Additionally, the impact of various substances, such as microbes, nutrients, and nucleotides, on the immune system remains an area ripe for discovery. Notably, the interplay between metabolism and immunity has garnered significant attention in recent years. Clarifying the roles of these substances within immune cells and discussing their therapeutic implications could lead to novel strategies for protecting against pathogens, controlling inflammation, and improving immunotherapy efficacy.

Moreover, the influence of other systems on the immune system is being increasingly recognized. For example, the bidirectional communication between the peripheral nervous system and the immune system is critical for mounting balanced and effective responses to invading pathogens. Neuro‐immune crosstalk is now understood to be a key mediator of immune function, potentially playing a role in immune dysregulation. Comprehensive understanding of how neurons regulate immune cell responses in barrier tissues and peripheral organs could unveil new therapeutic targets for a variety of diseases.^254^ In the hematological context, recent findings have highlighted the regulatory roles of erythroid cells in immune responses, particularly through their effects on immune cell activation and proliferation.^255^ These insights suggest that we must broaden our investigative scope beyond the immune system itself to include the interactions and influences of other bodily systems. Exploring these intersystem interactions could yield transformative insights and therapeutic advancements.

### 7.2 Opportunities for therapeutic interventions

Existing breakthroughs in immunotherapy have focused on three areas: autoimmune diseases, infections, and cancer. At the same time, these three areas are the major diseases currently affecting human health. Therefore, this section will focus on their possible future immunotherapy options.

#### 7.2.1 Treatment of autoimmune diseases and future perspectives

Up to now, there is no drug that can cure autoimmune diseases. Clinical treatment of autoimmune diseases mainly focuses on improving the condition, and therapeutic drugs include nonsteroidal anti‐inflammatory drugs, glucocorticoid, and antirheumatic drugs to improve the condition. All of these drugs have obvious side effects, and the probability of relapse is high after stopping the drugs. Recent research indicates that molecular therapies, such as monoclonal antibodies, B‐cell depleting agents, and CAR T cell treatments, can potentially benefit patients by addressing the limitations of traditional treatment methods.^256^ These therapies are a step closer to precision treatment, not only with fewer side effects, but also with the promise of a complete cure for autoimmune diseases.

Rituximab, a leading anti‐B‐cell drug, is a monoclonal antibody with both human and murine elements, targeting CD20. It depletes B cells in circulation, but its effects on B cells within the bone marrow, lymph nodes, and thymus are still under research and need further.^257^ It was first approved for non‐Hodgkin's B‐cell lymphomas and later found effective in treating RA and other autoimmune conditions affecting the central and peripheral nervous systems.^258^ Monoclonal antibodies target various components, with complement being a notable example. Complement receptors are present on several immune cell types, including B and T cells and DCs.^259^ Inhibition of complement activation therefore has an effect on T and B cell subsets in patients.^260^ Eculizumab, the first humanized monoclonal antibody targeting the complement system, prevents the cleavage of C5 into C5a and C5b, thereby blocking the formation of C5b‐9 MACs.^261^ Approved by the US FDA in 2007 for paroxysmal nocturnal hemoglobinuria, it is also used for atypical hemolytic uremic syndrome, systemic myasthenia gravis, and neuromyelitis optica.^262^ While CAR‐T cell therapy is mainly used for blood cancers, it shows potential for autoimmune diseases.^263^ Descartes‐08, an RNA‐based CAR‐T cell therapy, demonstrated positive results in a clinical trial with 14 adult patients with systemic myasthenia gravis.^264^

Autoimmune diseases result from the failure of mechanisms that maintain tolerance and distinguish between self and nonself. In the past decade, improvements in cellular phenotyping technology have significantly enhanced the resolution of immune phenotypes.^265^ These advancements can support future research by allowing more precise characterization of the genetic and genomic structures of different diseases. A deeper understanding of disease can aid in drug development as well as precision therapy. Currently, significant expectations are placed on molecular therapies, with research and development emphasizing drug safety, the potential for disease modification, steroid‐sparing capabilities, rapid onset of therapeutic effects, treatment duration, and patient adherence to prescribed administration routes.^256^

#### 7.2.2 Vaccine development for infectious diseases

Vaccines are the most favorable therapeutic weapon against infectious diseases. Traditional vaccines include inactivated and attenuated vaccines. The new generation of vaccines developed using molecular techniques includes recombinant protein vaccines, nucleic acid vaccines and viral vector vaccines. Of these, recombinant protein vaccines and traditional vaccines are the most widely used because of their recognized safety, stability and ease of manufacture. The advantage of viral vector vaccines is their ability to induce a strong and long‐lasting immune response. Viral vectors currently in use include adenovirus, retrovirus, lentivirus, and poxvirus. Of these, adenoviral vectors are the most commonly used and have been involved in the development of vaccines for various diseases such as Ebola, HIV, influenza, and SARS‐CoV‐2 (Table 5).^266^

**TABLE 5:** Novel vaccine strategies with their advantages and challenges.^236^

Vaccine type	Target pathogen	Mechanism of action	Advantages	Challenges
Nucleic acid	Influenza, COVID‐19, HIV, Zika virus, Ebola virus	Introduction of viral genetic material into cells to produce antigens using cellular transcription‐translation platforms	Higher stability, no risk of genomic integration and infectious origin, shorter preparation cycles	Needs to be transported at very low temperatures, safety and efficacy need to be clinically proven
Synthetic peptide	Allergy, HIV, cancer	Preparation of peptides with specific antigenic epitopes in pathogens by chemical synthesis techniques	Flexible design, fast production, high safety and easy quality control	The immune response is relatively weak and the effectiveness of synthetic peptide vaccines may be compromised if antigenic fragments change significantly.
Virus‐like particle	HBV, HPV, dengue virus	Hollow particles containing one or more structural proteins of a particular virus, without viral nucleic acids, which do not replicate autonomously but are morphologically identical or similar to true virus particles	Highly effective in inducing an immunoprotective response in humans and has an adjuvant effect	Complex and expensive manufacturing process
Protein subunit	Tuberculosis, HBV, HPV, respiratory syncytial virus, Herpes Zoster, COVID‐19, Pertussis vaccine	Extraction of special protein structures of bacteria and viruses, and screening of immunologically active fragments from them to make vaccines	Low inoculation side‐effects, fast antibody rise, long maintenance time, excellent immunological effect, and higher biological safety at the same time	Inability to express antigens intracellularly and thus be presented by MHC I, and therefore unable to activate T cells for killing
Vector‐based	MERS‐CoV, Poxvirus, yellow fever	Uses viral vectors to deliver antigen	Strong immune response, broad applicability, long‐term immune protection, high stability	Higher cost, abnormal immune responses due to mutation or inappropriate transmission of pathogens and need for specific storage conditions

Of the above vaccine types, the messenger RNA (mRNA) vaccine is currently the most popular subject of research. Indeed, mRNA vaccines utilize somatic cells to achieve posttranslational modification and complete function of proteins.^267^ In contrast to DNA vaccines, mRNA vaccines mitigate the risk of insertional mutagenesis in the host genome and can modulate antigen expression.^268^ In addition, mRNA vaccines can take advantage of the high yield of in vitro transcriptional reactions to achieve rapid development and mass production. To date, mRNA vaccines have been tested in preclinical and clinical trials in a variety of infectious diseases, including SARS‐CoV‐2, Zika virus, human immunodeficiency virus, influenza virus, cytomegalovirus, respiratory syncytial virus, varicella zoster virus, and rabies virus.^269^ Among these, the mRNA vaccine against SARS‐CoV‐2 virus has been approved for marketing.

Although good prospects for the use of mRNA vaccines can be foreseen, making them an alternative to traditional vaccines remains a challenge. Researchers are still searching for a suitable adjuvant and nano delivery vehicles. The right amount of effective adjuvant can promote innate and adaptive immunity and effectively assist the efficacy of vaccines.^243^ On the other hand, the safety of mRNA vaccines is yet to be proven. It is necessary to wait for the emergence of evidence from large‐scale clinical trials. In addition, mRNA vaccines may be less durable against viruses than other vaccines due to their different mechanisms. Therefore, mRNA vaccines need to be further developed and refined.

The COVID‐19 epidemic has once again impressed people with the great impact of infectious diseases on society. Factors such as increased globalization, increased climate change and the massive use of antibiotics all lead to faster mutation of pathogens and increased infectiousness. In the postepidemic era, the importance of vaccines is increasing day by day, and how to create vaccines with low cost, short production cycles, stable and long‐lasting preventive effects, and few side effects is a key direction of current research.

#### 7.2.3 Future direction for cancer treatment

The regulation of the immune system, including both suppression and activation, is crucial in cancer development. Therefore, immunotherapy is an important direction in cancer treatment. It aims to activate immune cells and overcome immune escape mechanisms.^270^ The current mainstream directions include immune checkpoint blockade (ICB) and ACT therapies, and there has also been some research progress in tumor vaccines.^271^ Established studies have clearly recognized the heterogeneity of TME, and immunotherapy is able to kill cancer cells in a more personalized and precise manner and produce longer‐lasting efficacy compared with existing therapeutic techniques. ICs are inhibitory molecules found on various immune cells that play a crucial role in cancer immune evasion.^272^ Currently, the targets of ICB are mainly focused on T cells, which have the most direct killing effect on tumors. PD‐1/PD‐L1 and CTLA‐4/B7‐1/2 were the first IC pathways to be investigated, and researchers have since identified targets such as Tim‐3, LAG‐3, NR2F6, TIGIT, V‐type immunoglobulin domain‐containing suppressor of T cell activation (VISTA), and B‐ and T‐lymphocyte attenuator (BTLA).^270^ Currently, ICB therapy has gained some efficacy in NSCLC, colon cancer, melanoma and RCC,^273^ but the shortcoming is that only 20−30% of patients respond well to the treatment.^274^ This may be due to the presence of other inhibitory molecules expressed within the patient's tumor. Therefore, the focus of future ICB therapy should be centered on the discovery of new IC targets as well as biomarkers to predict clinical efficacy.

Cellular immunotherapies mainly include CAR T cell therapy, tumor‐infiltrating lymphocytes (TILs) therapy, engineered TCR therapy, and NK‐cell therapy, with TIL therapy being the first to be introduced. TILs are heterogeneous populations of T cells that infiltrate within tumors. In most cancer patients, the amount of spontaneously produced TIL is too small and therefore insufficient to inhibit cancer growth. TIL therapy involves isolating this population of cells from the tumor, using IL‐2 stimulation and thus expanding them in an in vitro laboratory setting, and finally re‐injecting them into the patient.^275^ TIL therapy has been shown to have significant therapeutic effects in solid tumors such as ovarian cancer, osteosarcoma, metastatic breast cancer and melanoma.^270^ More clinical studies are needed in the future to expand TIL applications to other cancer scenarios. Engineered TCR therapy is similar in principle to CAR T cell therapy, but CAR recognizes tumor‐associated antigens independently of MHC presentation, thus freeing CAR T cell therapy from MHC constraints and enabling a more direct tumor‐killing effect.^276^ CAR‐T cell therapy has now gained good clinical application in hematological tumors, but the therapeutic effect on solid tumors still needs to be further strengthened. In addition, the problem that needs to be solved urgently is the serious side effects and fatal toxicity caused by CAR T cells after entering the body, including cytokine storm, immune effector cell‐associated neurotoxicity syndrome, and so on.^277^

CAR NK‐cell therapy is almost identical to CAR T cell therapy except that the editing target is NK cells. CAR NK‐cell therapy is almost identical to CAR T cell therapy except that the editing target is NK cells. However, its ability to expand in vivo is much less than that of T cells, so the durability of this therapy is one of the future research directions.^278^ In addition, it is also a challenge to enhance the cytotoxicity of NK cells due to their susceptibility to TME.^279^

## 8 CONCLUSION

This review begins with a brief overview of the components of innate immunity, including barrier structures, cellular and humoral components. It then proceeds to discuss the functions of innate immunity, including recognition of pathogens, initiation of inflammatory responses, and the recently discovered concept of trained immunity. Having gained an understanding of innate immunity, we proceeded to introduce key components of adaptive immunity, namely, T cells, B cells, and antibodies, and discussed their roles in cellular immunity, humoral immunity and immunological memory. Having established the fundamentals of innate immunity, we then show the crosstalk between innate and adaptive immunity, which focus on the participation of innate immunity in adaptive immunity, the cytokine signaling, and the maintenance of immune homeostasis. The significance of studying the interaction between innate and adaptive immunity lies in its potential to revolutionize our approach to disease treatment. Therefore, we explored how this interplay affects the pathogenesis of infectious diseases, autoimmune diseases, and cancer, providing insights into therapeutic strategies. Targeting innate immune pathways and enhancing adaptive immune responses are crucial in immunomodulatory drug development, and innovative vaccine strategies leverage these interactions for better efficacy.

Understanding the interaction between innate and adaptive immunity is vital. It not only clarifies the traditional distinctions and connections between these two branches but also guides the development of immunotherapies in multiple aspects, levels and depths. More importantly, this review prompts a reevaluation of the boundaries between innate and adaptive immunity, encouraging the exploration of new immunological concepts and advancing immunological research. Future research should continue to explore the precise mechanisms regulating the interactions between innate and adaptive immunity, particularly those involving signaling pathways, cytokines, and immune cell interactions. Additionally, it is necessary to develop and optimize therapies targeting both innate and adaptive immune components, such as combination therapies involving checkpoint inhibitors, CAR‐T cells, and innate immune activators. Furthermore, it is important to Investigate the potential of immunomodulatory agents to enhance the efficacy of existing treatments and overcome resistance mechanisms, especially in the context of the TME and chronic infections. Finally, leveraging advanced technologies like single‐cell multiomics and CRISPR‐based gene editing can dissect the complexities of immune interactions and identify novel therapeutic targets.

In conclusion, a deeper exploration of the interaction between innate and adaptive immunity offers exciting opportunities to enhance our understanding of the immune system and to develop optimized immunotherapeutic strategies. As technology advances and the complexity of the immune system is better understood, we anticipate more breakthroughs in immunology, leading to more effective and personalized disease prevention and treatment.

## AUTHOR CONTRIBUTIONS

Ruyuan Wang and Caini Lan wrote the article and drew the figures. Kamel Benlagha, Niels Olsen Saraiva Camara, Heather Miller, Masato Kubo, Pamela Lee, Huamei Forsman, Lu Yang, and Steffen Heegaard reviewed the paper. Xingrui Li, Zhimin Zhai, and Caini Lan organized and revised the paper. All authors contributed to the article and approved the submitted version.

## CONFLICT OF INTEREST STATEMENT

We declare that the research was conducted in the absence of any commercial or financial relationships that could be construed as a potential conflict of interest.

## ETHICS STATEMENT AND CONSENT TO PARTICIPATE

Not applicable.

## Data Availability

Data availability is not applicable to this review as no new data were created or analyzed in this review.
